# Synthesis of 1-azaspiro[4.4]nonan-1-oxyls via intramolecular 1,3-dipolar cycloaddition

**DOI:** 10.3762/bjoc.15.200

**Published:** 2019-08-27

**Authors:** Yulia V Khoroshunova, Denis A Morozov, Andrey I Taratayko, Polina D Gladkikh, Yuri I Glazachev, Igor A Kirilyuk

**Affiliations:** 1 N.N. Vorozhtsov Institute of Organic Chemistry SB RAS, Academician Lavrentiev Ave. 9, Novosibirsk 630090, Russian Federation; 2 Novosibirsk State University, Pirogova Str. 2, Novosibirsk 630090, Russian Federation; 3 Voevodsky Institute of Chemical Kinetics and Combustion SB RAS, Institutskaya 3, Novosibirsk 630090, Russian Federation

**Keywords:** aldonitrone, 1,3-dipolar cycloaddition, pyrrolidine nitroxides, 1-pyrroline-*N*-oxide, sterically shielded nitroxide

## Abstract

Sterically shielded nitroxides of the pyrrolidine series have shown the highest resistance to reduction. Here we report the synthesis of new pyrrolidine nitroxides from 5,5-dialkyl-1-pyrroline *N*-oxides via the introduction of a pent-4-enyl group to the nitrone carbon followed by an intramolecular 1,3-dipolar cycloaddition reaction and isoxazolidine ring opening. The kinetics of reduction of the new nitroxides with ascorbate were studied and compared to those of previously published (1*S*,2*R*,3′*S*,4′*S*,5′*S*,2″*R*)-dispiro[(2-hydroxymethyl)cyclopentan-1,2′-(3′,4′-di-*tert*-butoxy)pyrrolidine-5′,1″-(2″-hydroxymethyl)cyclopentane]-1′-oxyl (**1**).

## Introduction

**Figure 1:**
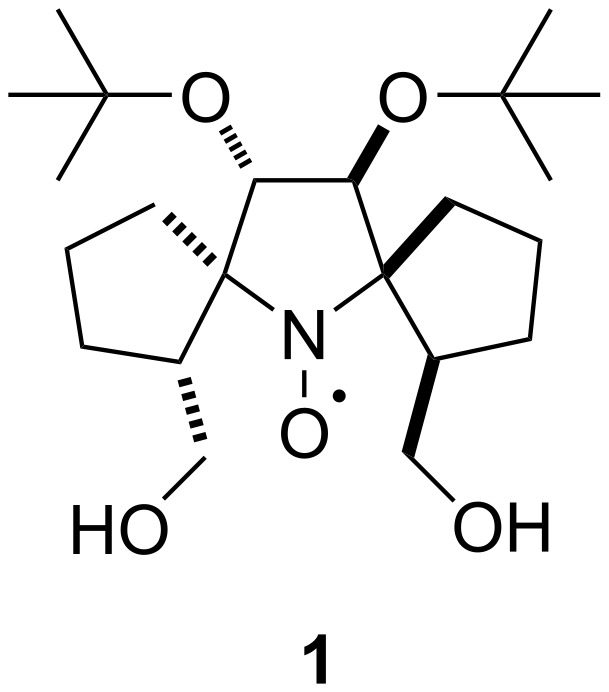
Structure of nitroxide **1**.

Sterically shielded nitroxides are currently attracting much attention due to their high resistance to bioreduction [1–2]. It has been demonstrated that 2,2,5,5-tetraethylpyrrolidine nitroxides have the highest stability, sometimes exceeding that of trityl radicals [1]. Introduction of spirocyclic moieties has a smaller effect on the reduction rates of nitroxides than the introduction of linear alkyl substituents does; however, spirocyclic nitroxides may have much longer spin relaxation times at 70–125 K [3] and even at room temperature [4]. The latter effect may be useful for structural studies by means of PELDOR or DQC [5]. We recently reported the synthesis of sterically shielded pyrrolidine nitroxide **1** via a stereospecific consecutive assembly of two spiro-(2-hydroxymethyl)cyclopentane moieties. These procedures included the addition of pent-4-enylmagnesium bromide to the corresponding nitrone, oxidation to alkenylnitrone, intramolecular 1,3-dipolar cycloaddition, and isoxazolidine ring opening. Nitroxide **1** showed both an unexpectedly low reduction rate [6] and long relaxation times *t*_1_ and *t*_m_ at room temperature [4]. Unexpectedly high resistance of this nitroxide to chemical reduction results from the configuration of the hydroxymethyl groups, which are directed towards the nitroxide group, thereby making it more hindered. It is known that inductive effects of substituents can strongly affect the rate of nitroxide reduction [7–8]; therefore, one could expect that the removal of electron-withdrawing *tert*-butoxy groups at positions 3 and 4 of the pyrrolidine ring of **1** (Figure 1) should lead to a further decrease in the reduction rate of the nitroxide.

Here we describe the synthesis of *C*_1_-symmetric racemic 3,4-unsubstituted pyrrolidine nitroxides with only one spiro(2-hydroxymethyl)cyclopentane moiety. The rates of reduction of the new nitroxides with ascorbate were measured.

## Results and Discussion

**Scheme 1:**
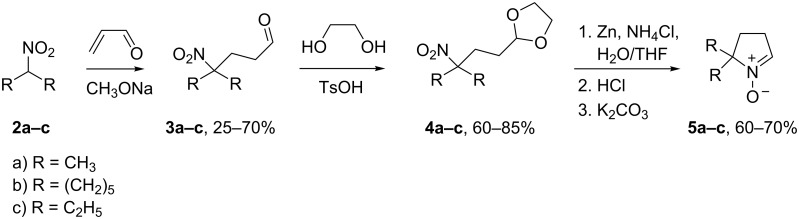
The synthesis of aldonitrones **5a–c**.

Aldonitrones **5b**,**c** were prepared similarly to the well-known synthesis of 5,5-dimethyl-1-pyrroline-1-oxide (DMPO, **5a**) [9–10] from nitrocyclohexane and 3-nitropentane (Scheme 1). In brief, the reaction of nitrocyclohexane and acrolein in a CH_3_ONa/CH_3_OH solution afforded the corresponding nitroaldehyde **3b** with a 70% yield. Of note, the Michael addition of 3-nitropentane to acrolein was accompanied by remarkable tarring and gave a much lower yield of nitroaldehyde **3c** (25%). The reactive aldehyde groups were protected via 1,3-dioxolane assembly, and the resulting compounds were treated with Zn dust and NH_4_Cl in a water–THF solution to reduce nitro groups. The resulting hydroxylamines were treated with hydrochloric acid to hydrolyse dioxolane moieties, and careful basification resulted in intramolecular cyclisation givnig **5b**,**c** with a yield of 51% and 42%, respectively.

**Scheme 2:**
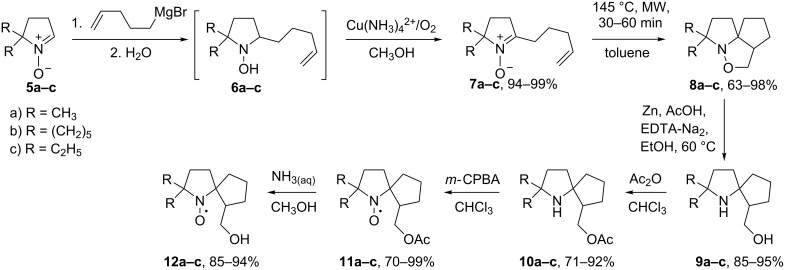
The principal synthetic scheme for nitroxides **12a–c**.

Nitrones **5a–c** readily react with 4-pentenylmagnesium bromide. Quenching of the reaction mixtures with water under aerobic conditions leads to partial oxidation of resultant *N*-hydroxypyrrolidines **6a–c** to corresponding nitrones **7a–c**. Therefore, this conversion was finalised via bubbling of air into the solution in the presence of Cu(NH_3_)_4_^2+^ (Scheme 2).

**Scheme 3:**
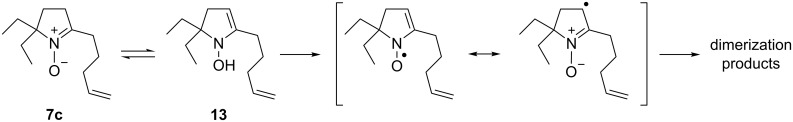
A possible pathway of ketonitrone **7c** self-transformations.

Samples of resulting 2-(pent-4-enyl)nitrones **7a–c** remarkably deteriorate during storage under aerobic conditions with dark tar formation. A possible pathway of decay may include oxidation of the ene-hydroxylamine tautomeric form to vinylnitroxide; similar compounds are prone to various dimerisation reactions (Scheme 3) [11]. It is worth noting that in the mass spectrum of **7c**, the [M − 1]^+^ ion was observed instead of the molecular ion. The easy loss of a hydrogen atom is consistent with the susceptibility of **7c** to oxidative decay (Scheme 3).

Intramolecular cycloaddition of similar nitrones is known to lead to hexahydro-1*H*-cyclopenta[*c*]isoxazoles [6,12]. Indeed, heating of **7а–с** at 145 °C in toluene for 30–60 min in a microwave oven produced **8a–c** (racemic mixtures; Scheme 2). The structure assignment was performed on the basis of ^1^H and ^13^C NMR spectra and ^1^H,^1^H and ^13^C,^1^H correlations (see Supporting Information File 1); the spectral data are in agreement with the literature on similar systems [6].

To decrease the tarring, the reaction was carried out in the presence of 2,2,6,6-tetramethylpiperidine-1-oxyl. It should be noted that heating of alkenyl nitrones **7a** and **7b** gives the corresponding cycloadducts with yields close to quantitative, whereas for nitrone **7c**, the complete conversion could not be achieved either at 145 °C or at a higher temperature. According to the ^1^Н NMR spectra, the **8c**/**7c** ratio never exceeded 3:1. Heating of a pure sample of **8c** under similar conditions caused the emergence of signals at 5.70, 4.92, and 4.87 ppm in the ^1^H NMR spectra. These signals were attributed to the protons of the terminal vinyl group of **7c**. 1,3-Dipolar cycloaddition of nitrones to alkenes is known to be reversible [13–14]. We recently reported a similar reversibility of the intramolecular cyclization of sterically hindered pent-4-enylnitrone of the 2*H*-imidazole series [15].

Treatment with Zn in an AcOH/EtOH/EDTA/Na_2_ mixture was performed for reductive isoxazolidine ring opening [15–16] producing aminoalcohols **9a–c** in 85–95% yields (Scheme 2).

**Scheme 4:**
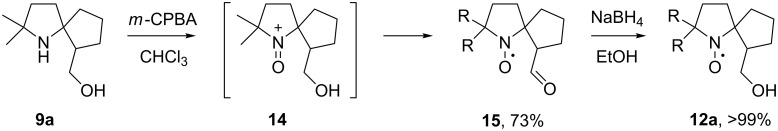
Oxidation of aminoalcohol **9a**.

We have previously reported that oxidation of secondary amines with a spiro(2-hydroxymethyl)cyclopentane moiety at the α-carbon with the H_2_O_2_/WO_4_^2−^ system is ineffective whereas conversion of these amines to the corresponding nitroxides can be easily performed using *m*-chloroperbenzoic acid (*m*-CPBA) [6,15]. Treatment of **9a** with *m*-CPBA in dry chloroform at −10 °C afforded a nitroxide, which was isolated as orange oil with a yield of 73% (Scheme 4). An infrared spectrum of the isolated compound showed a strong absorption band at 1725 cm^−1^ typical for carbonyl compounds and no absorption in the region 3100–3500 cm^−1^, suggesting that the hydroxymethyl group was affected in the reaction. The mass spectrum featured the molecular ion [M^+^] = 196.1335 corresponding to the molecular formula C_11_H_18_NO_2_, which matches element analysis data. These results allowed us to assign the structure **15** to this nitroxide. Indeed, oxidation of amines with peracids is known to proceed via oxoammonium cation formation [17–18], and the latter can oxidize alcohols to carbonyl compounds [19]. The close proximity of the hydroxymethyl group to the oxoammonium one favours the reaction. Treatment of **15** with NaBH_4_ in EtOH caused quantitative reduction of the aldehyde group to the hydroxymethyl one, thus yielding **12a** identical to that prepared by the alternative method (see below).

To prevent oxidative reactions in the side chain, the hydroxymethyl group in **9a–c** was protected via acylation. Heating of **9a–c** with an excess of acetic anhydride in chloroform quantitatively afforded the corresponding esters **10a–c**. The products of acylation of the sterically hindered amino group were not detected in the reaction mixture.

**Scheme 5:**
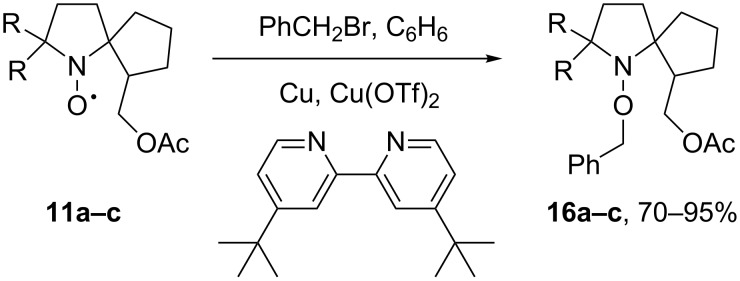
The synthesis of alkoxyamines **16a–c**.

Oxidation of **10а–c** with *m*-CPBA yielded the desired nitroxides **11a–c** as orange oils. IR spectra of the new nitroxides contained no absorption bands in the region of 3000–3600 cm^−1^ and did not have an intense band at 1740 cm^−1^ characteristic for the ester C=O group. To confirm the structure of the nitroxides, alkoxyamines **16a–c** were prepared by Matyjaszhewski's method (Scheme 5) [20].

**Scheme 6:**
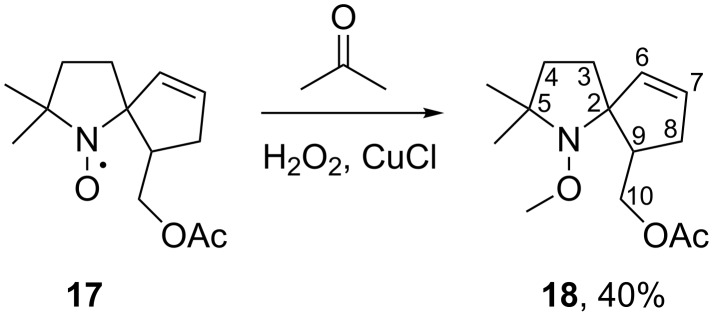
The alkoxyamine **18** synthesis.

Oxidation of aminoacetate **10a** along with the expected formation of nitroxyl radical **11a**, gave another nitroxide, **17**, in 22% yield. The IR spectra of **11a** and **17** are very similar, showing the bands typical for ester group vibrations and no bands that could be attributed to the vibrations of OH or NH groups. Besides that in the spectrum of compound **17**, there are absorption bands near 3054 and 1620 cm^−1^, which denote the presence of a double C=C bond. To elucidate the structure, nitroxyl radical **17** was converted into alkoxyamine **18** (Scheme 6) using the literature method by Schoening et al. [21], and ^1^H and ^13^C NMR spectra, as well as two-dimensional ^1^H,^1^H-COSY and ^1^H,^13^C-HSQC and HMBC spectra were recorded (see Supporting Information File 1). Signals at 4.14 and 4.45 ppm in the ^1^H NMR spectrum were assigned to the hydrogen atoms of the O–C(10)H_2_ group. Analysis of the ^1^H,^1^H-COSY and ^1^H,^13^C-HSQC NMR spectra allowed us to unambiguously assign a signal at 2.26 ppm in the ^1^H NMR spectrum to the methine proton on the C(9) carbon atom (see Supporting Information File 1). This signal in the ^1^H,^1^H-COSY spectrum contains two cross-peaks with hydrogens of the O–C(10)H_2_ group and two additional cross-peaks with signals at 2.11 and 2.36 ppm, which were assigned to the C(8)H_2_ group. The chemical shifts and character of the splitting for this group of signals correspond to structural fragment O–C(10)H_2_–C(9)H–C(8)H_2_. The signals of the C(8)H_2_ group in the COSY spectrum contain only two additional cross-peaks with the olefin signals at 5.64 and 5.86 ppm. Analysis of the ^1^H,^13^C-HSQC spectrum revealed that the latter protons are bound to carbon atoms with chemical shifts 135.3 and 131.1 ppm, respectively, and this finding enabled the assignment of these signals to the 1,2-disubstituted alkene moiety. Thus, the NMR data presented above indicate the presence of an isolated spin system, O–CH_2_–CH–CH_2_–CH=CH. A similar analysis of the remaining complex multiplets at 1.56, 1.77, and 1.93 ppm as well as their correlation with the signals of carbon atoms in the ^1^H,^13^C-HSQC spectra allowed us to assign these signals to an isolated CH_2_–CH_2_ system. All these findings unambiguously support the assignment of structure **18** to the isolated alkoxyamine and structure **17** to the corresponding nitroxide.

**Scheme 7:**
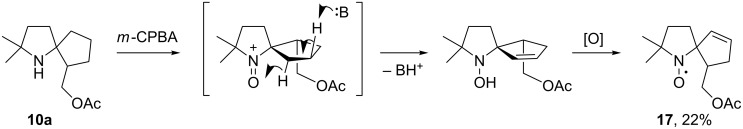
**A** possible mechanism of nitroxide **17** formation.

Therefore, **17** is formed due to hydrogen abstraction in the spirocyclopentane ring. To the best of our knowledge, similar transformations have never been observed before. Presumably, formation of **17** occurs due to the close proximity of the N^+^=O group (in the intermediate strained oxoammonium cation) to the hydrogen atom of an adjacent methylene group of the cyclopentane ring (Scheme 7).

**Scheme 8:**
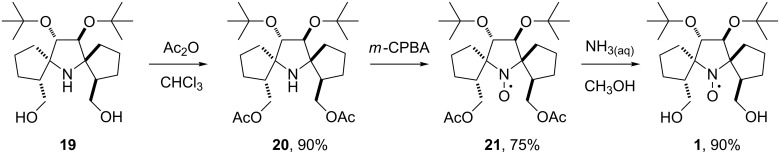
Optimisation of the synthesis of nitroxide **1**.

The ester groups in **11a–c** were easily cleaved in an aqueous–methanol solution of ammonia. Nitroxides **12a–c** were isolated as orange compounds moderately soluble in water. Overall yields of these radicals via the acylation–oxidation–deprotection pathway were in the range of 54–75%. Of note, the use of a similar three-step procedure for the synthesis of **1** from **19** increased the yield of this nitroxide from 20% [6] to 60% (Scheme 8).

The EPR spectra of nitroxides **12a–c** and **1** acquired in a deoxygenated buffer revealed a significant difference in line widths (see Table 1 and Supporting Information File 1, Figures S7–S10), with the broadest lines expectedly being shown by **12b**. Introduction of spirocyclohexane moieties to α-carbons of pyrrolidine nitroxides was found to cause strong broadening of lines in the EPR spectra, presumably owing to unresolved *hfc* on the hydrogens at positions 2 and 6 of the cyclohexane ring [22]. It has been reported that EPR spectra of pyrrolidine or imidazolidine nitroxides with pair(s) of geminal ethyl groups at α-carbon atoms may feature large doublet hyperfine splittings [23–25]. For imidazolidine nitroxides, these splittings were unambiguously attributed to *hfc* on one of four methylene hydrogens of each pair of ethyls [23]. The difference in apparent hyperfine coupling constant *a*_H_ on these hydrogens is due to a substituent at position 3 or 4 of the ring. This substituent hinders rotation and affects the population of conformations of neighbouring geminal ethyl groups, thereby preventing averaging. In agreement with this conclusion, there are no large splittings in the EPR spectrum of **12c**, and the line width is a bit greater than that in the spectra of similar nonspirocyclic 3,4-unsubstituted 2,2-diethylpyrrolidine nitroxides [26], implying free rotation of ethyl groups. In the EPR spectra in toluene the nitroxides **11a–c** show 0.035–0.04 mT lower *hfs* constants on nitrogen atom compared to nitroxides **12a–c**, presumably due to intramolecular hydrogen bond formation. The difference in *a*_N_ between **11a–c** and **12a–c** is almost one order of magnitude lower for EPR spectra in water (0.005–0.006 mT), obviously because strong intermolecular hydrogen bonds are formed due to solvation.

**Table 1:** Parameters of the EPR spectra (hyperfine coupling constants, *a*_N_; peak-to-peak linewidths, *ΔB_p-p_*; and *g*-factors), second order rate constants of reduction with ascorbate and partition coefficients octanol-water (*K*_p_) for nitroxides **1** and **12a–c**.

Nitroxide	*a*_N_, mT	Δ*B_p-p_*, mT	*g*-factor	*k_2_*, M^−1^s^−1^	*K*_p_

**1**	1.481	0.29	2.00553(±2)	(3.6 ± 0.2) × 10^−3^	600
**12a**	1.595	0.21	2.00549(±2)	(7.3 ± 0.2) × 10^−2^	12
**12b**	1.586	0.34	2.00553(±2)	(4.6 ± 0.5) × 10^−2^	130
**12c**	1.570	0.24	2.00552(±2)	(2.2 ± 0.4) × 10^−2^	80

**Figure 2:**
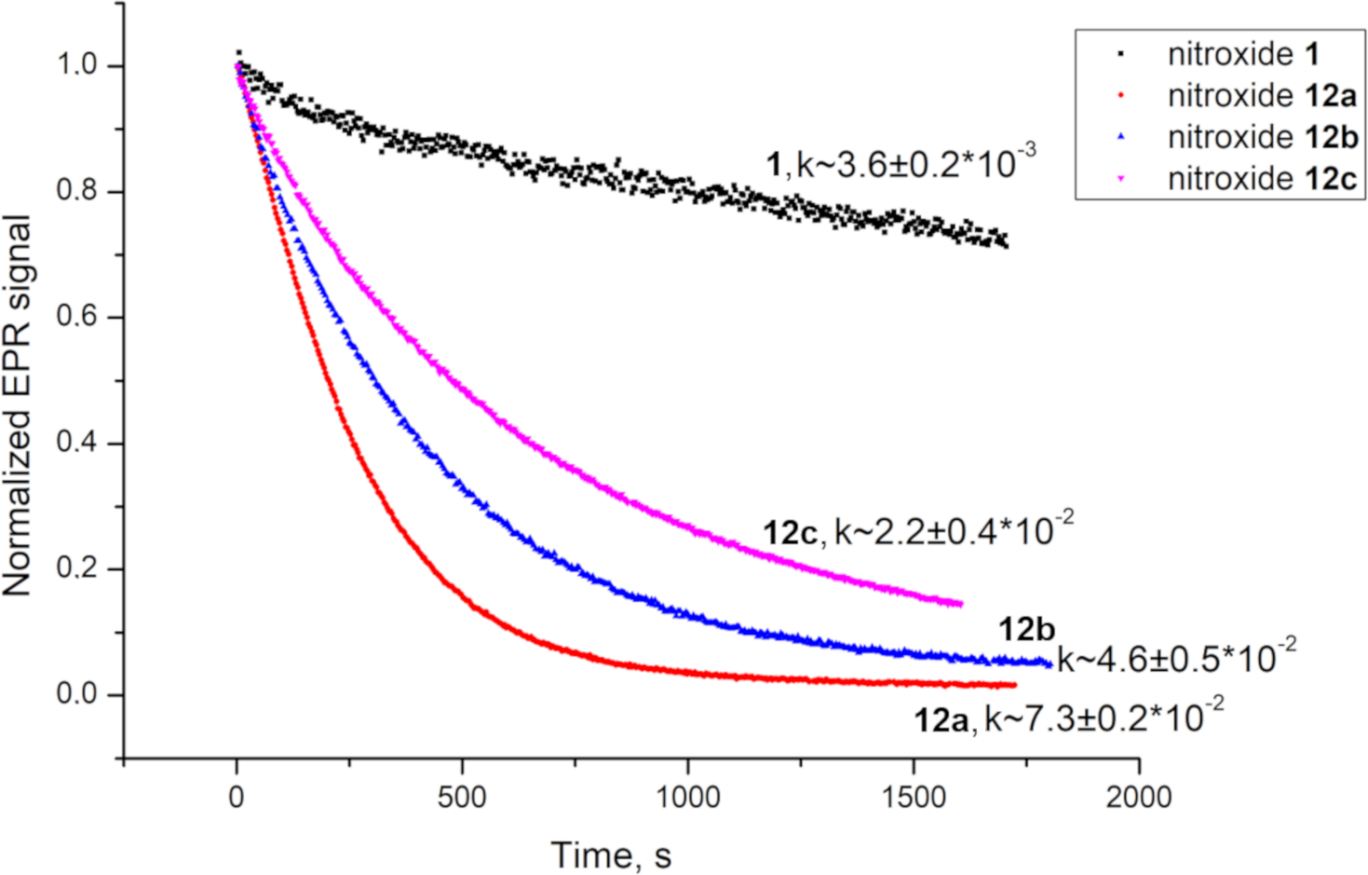
Kinetics of the reduction of nitroxides **1** and **12a–c** (0.3 mM) with ascorbate (50 mM) in 50 mM phosphate-citrate-borate buffer in the presence of glutathione (2 mM), at pH 7.4 and, temperature 293 K. Second-order rate constants, *k* (M^−1^s^−1^), for the initial rates of reduction are presented.

The initial rates of the EPR signal decay were used to obtain the rate constants of the nitroxide reduction by ascorbic acid (see Figure 2 and Supporting Information File 1).

The rate constants for all these nitroxides are remarkably higher than this constant for nitroxide **1**. Even nitroxide **12c**, which is the most stable among the new pyrrolidine nitroxides has a ca. 6-fold higher reduction rate than **1** does, and both are less resistant to reduction than 2,2,5,5-tetraethyl-substituted pyrrolidine nitroxides are (*k* = 2 × 10^−3^ to 3.3 × 10^−4^ ) [1,25]. The rate of reduction of **12a** is close to that of 3-carboxy-2,2,5,5-tetramethylpyrrolidine-1-oxyl [24]. Obviously, a single spiro-(2-hydroxymethyl)cyclopentane moiety cannot provide higher reduction resistance than geminal ethyl groups can. Thus, estimation of the steric effect of neighbouring substituents cannot account for the high reduction resistance of **1**. Presumably, the symmetric structure with bulky substituents at positions 3 and 4 is an important factor for nitroxide stability. Due to the steric repulsion of *trans*-oriented *tert*-butoxy groups and spiro cyclopentane moieties in the symmetric structure of **1**, the (2-hydroxymethyl)cyclopentane groups tightly embrace the nitroxide group making it less accessible for reductants. It is also noteworthy that the symmetric repulsion from both sides of the pyrrolidine ring favours a planar nitroxide group and destabilises the corresponding hydroxylamine with the sp^3^-hybridised nitrogen. Recently, we observed a similar effect for (3*S*(*R*),4*S*(*R*))-2,2,5,5-tetraethyl-3,4-bis(hydroxymethyl)pyrrolidine-1-oxyl, which manifested the highest resistance to reduction among known nitroxides [25]. In contrast, structures **12a–c** are asymmetric, and corresponding hydroxylamines could be additionally stabilised by hydrogen bonding between the nitrogen atom and the proton of the hydroxymethyl group.

## Conclusion

In this study, we again demonstrated feasibility of the general synthetic approach to sterically hindered spirocyclic nitroxides based on an intramolecular 1,3-dipolar cycloaddition reaction in alkenylnitrones followed by isoxazolidine ring opening. The resulting asymmetric pyrrolidine nitroxides have unexpectedly high rates of reduction with ascorbate. These results lead us to the assumption that symmetric structures with bulky substituents at positions 3 and 4 should be favoured for achieving higher resistance to reduction.

## Supporting Information

File 1 Full experimental details and analytical data (UV, IR, ^1^H NMR, ^13^C NMR, and EPR experiments, and microanalysis).

## References

[1] Jagtap A P, Krstic I, Kunjir N C, Hänsel R, Prisner T F, Sigurdsson S T (2015). Free Radical Res.

[2] Sasaki K, Ito T, Fujii H G, Sato S (2016). Chem Pharm Bull.

[3] Kathirvelu V, Smith C, Parks C, Mannan M A, Miura Y, Takeshita K, Eaton S S, Eaton G R (2009). Chem Commun.

[4] Kuzhelev A A, Strizhakov R K, Krumkacheva O A, Polienko Y F, Morozov D A, Shevelev G Y, Pyshnyi D V, Kirilyuk I A, Fedin M V, Bagryanskaya E G (2016). J Magn Reson.

[5] Meyer V, Swanson M A, Clouston L J, Boratyński P J, Stein R A, Mchaourab H S, Rajca A, Eaton S S, Eaton G R (2015). Biophys J.

[6] Morozov D A, Kirilyuk I A, Komarov D A, Goti A, Bagryanskaya I Y, Kuratieva N V, Grigor’ev I A (2012). J Org Chem.

[7] Morris S, Sosnovsky G, Hui B, Huber C O, Rao N U M, Swartz H M (1991). J Pharm Sci.

[8] Kirilyuk I A, Bobko A A, Semenov S V, Komarov D A, Irtegova I G, Grigor’ev I A, Bagryanskaya E (2015). J Org Chem.

[9] Haire D L, Janzen E G (1982). Can J Chem.

[10] Turner M J, Rosen G M (1986). J Med Chem.

[11] Aurich H G, Hahn K, Stork K (1979). Chem Ber.

[12] Dondas H A, Grigg R, Hadjisoteriou M, Markandu J, Thomas W A, Kennewell P (2000). Tetrahedron.

[13] Coşkun N (1997). Tetrahedron.

[14] Coşkun N, Tirli Tat F, Özel Güven Ö (2001). Tetrahedron.

[15] Edeleva M V, Parkhomenko D A, Morozov D A, Dobrynin S A, Trofimov D G, Kanagatov B, Kirilyuk I A, Bagryanskaya E G (2014). J Polym Sci, Part A: Polym Chem.

[16] Sár C P, Ősz E, Jekő J, Hideg K (2005). Synthesis.

[17] Sen V D, Golubev V A, Efremova N N (1982). Bull Acad Sci USSR, Div Chem Sci (Engl Transl).

[18] Toshimasa T, Eiko M, Keisuke M (1972). Bull Chem Soc Jpn.

[19] Bobbitt J M, Brückner C, Merbouh N (2009). Org React.

[20] Matyjaszewski K, Woodworth B E, Zhang X, Gaynor S G, Metzner Z (1998). Macromolecules.

[21] Dichtl A, Seyfried M, Schoening K-U (2008). Synlett.

[22] Kirilyuk I A, Polienko Y F, Krumkacheva O A, Strizhakov R K, Gatilov Y V, Grigor’ev I A, Bagryanskaya E G (2012). J Org Chem.

[23] Bobko A A, Kirilyuk I A, Gritsan N P, Polovyanenko D N, Grigor’ev I A, Khramtsov V V, Bagryanskaya E G (2010). Appl Magn Reson.

[24] Paletta J T, Pink M, Foley B, Rajca S, Rajca A (2012). Org Lett.

[25] Dobrynin S A, Glazachev Y I, Gatilov Y V, Chernyak E I, Salnikov G E, Kirilyuk I A (2018). J Org Chem.

[26] Lampp L, Morgenstern U, Merzweiler K, Imming P, Seidel R W (2019). J Mol Struct.

