# Supplementary material for: Synthesis of 1-azaspiro[4.4]nonan-1-oxyls via intramolecular 1,3-dipolar cycloaddition
Source: Beilstein J Org Chem. 2019 Aug 27;15:2036–42. doi: 10.3762/bjoc.15.200 (PMC6720653; doi:10.3762/bjoc.15.200)
Supplement: File 1 — Full experimental details and analytical data (UV, IR, 1H NMR, 13C NMR, and EPR experiments, and microanalysis). [file Beilstein_J_Org_Chem-15-2036-s001.pdf]

BEILSTEIN JOURNAL OF ORGANIC CHEMISTRY

Supporting Information

for

Synthesis of 1-azaspiro[4.4]nonan-1-oxyls via intramolecular 1,3-dipolar cycloaddition

Yulia V. Khoroshunova, Denis A. Morozov, Andrey I. Taratayko, Polina D. Gladkikh, Yuri I. Glazachev and Igor A. Kirilyuk

Beilstein J. Org. Chem. **2019,** *15,* 2036–2042. doi:10.3762/bjoc.15.200

Full experimental details and analytical data (UV, IR, ^{1}H NMR, ^{13}C NMR, and EPR experiments, and microanalysis)

License and Terms: This is a supporting information file under the terms of the Creative Commons Attribution License (http://creativecommons.org/licenses/by/4.0). Please note that the reuse, redistribution and reproduction in particular requires that the authors and source are credited.
The license is subject to the *Beilstein Journal of Organic Chemistry* terms and conditions: (https://www.beilstein-journals.org/bjoc)

Table of contents

1. Experimental section and NMR data for the new compounds..........................S2
2. EPR experiments...S27
3. References..S34

All commercially available solvents and reagents were of highest quality and used without further purification. Nitrone **5a** was prepared according to the literature protocols [1, 2]. ^{1}H NMR spectra were recorded at 300, 400, 500 or 600 MHz on BRUKER Avance AV 300, AV 400, DRX 500 and AV 600 spectrometers. ^{13}C NMR spectra were recorded at 75, 100, or 150 MHz on BRUKER Avance AV 300, AV 400 and AV 600 spectrometers, as indicated next to each NMR analysis. ^{1}H and ^{13}C chemical shifts (δ) were internally referenced to the residual solvent peak. The J values in ^{1}H NMR spectra are given in Hz. The IR spectra were acquired on a FTIR spectrometer in KBr or neat. The UV spectra were recorded in EtOH (10^{-4} M solutions). The progress of the reactions was monitored by TLC and carried out using UV light at 254 nm, 1% aqueous potassium permanganate, and/or Dragendorff reagent as visualizing agents. Column chromatography was performed on silica gel 60 (70−230 mesh). HRMS were recorded on double-focusing, high-resolution mass spectrometer equipped with a high-performance toroidal ESA and quadrupole time-of-flight mass spectrometer.

3-Nitropentane and nitrocyclohexane were prepared from pentan-3-one oxime and cyclohexanone oxime according to literature protocols [3].

Nitroaldehydes 3b,c (General procedure). A solution of nitroalkane **2b,c** (50 mmol) in CH_3OH (25 mL) was added dropwise to a freshly prepared solution of CH_3ONa (54 mg, 1 mmol) in CH_3OH (10 mL) at 10 °C. The resulting pale yellow solution was stirred at room temperature for 15 minutes and then cooled to 2 °C. Freshly distilled acrolein was added dropwise to the reaction mixture maintaining a temperature of 2–4 °C. Then AcOH (0.06 mL, 1 mmol) was added and CH_3OH was evaporated under reduced pressure. The crude residue was dissolved in $CHCl_3$ (50 mL), washed with brine (3 ×

40 mL) and dried with Na_2SO_4. After evaporation of the chloroform, the crude residue was purified via column chromatography (silica gel, $CHCl_3$).

3-(1-Nitrocyclohexyl)propanal (3b):

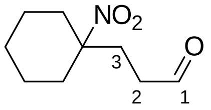

Yield : 70%, colorless liquid which solidifies when cooled to −20 °C.

Spectroscopic data are identical to previously published [4].

^{1}H NMR (500 MHz, $CDCl_3$): 1.17-1.36 (m, 3H)(cyclohexane); 1.42-1.57 (m, 5H)(cyclohexane); 2.02-2.07 (m, 2H)(C(3)H_2); 2.24-2.32 (m, 2H)(cyclohexane; 2.34-2.40 (m, 2H) (C(2)H_2); 9.64 (t, J_t=0.9, 1H)(HC=O).

^{13}C NMR (125 MHz, $CDCl_3$): 21.9, 24.3, 31.6, 33.7, 37.6, 90.2 (C-NO_2), 199.6 (HC=O).

4-Ethyl-4-nitrohexanal (3c):

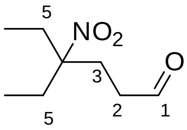

Yield : 25%, colorless liquid.

IR (neat) ν, cm^{-1}: 2977, 2946, 2887, 2836, 2730, 1726, 1535, 1456, 1388, 1358, 1311, 849, 827.

^{1}H NMR (500 MHz, $CDCl_3$): 0.83 (t, J_t=7.5, 6H)(2×CH_3); 1.83-2.03 (m, 4H)(C(5)H_2); 2.14-2.24 (m, 2H)(C(3)H_2); 2.36-2.44 (m, 2H)(C(2)H_2); 9.74 (t, J_t=1,1H)(HC=O).

^{13}C NMR (125 MHz, $CDCl_3$): 7.8 (2×CH_3), 26.6, 27.8, 38.1, 94.3 (C-NO_2), 199.7 (HC=O).

Anal. calcd for $C_8H_{15}NO_3$: C 55.47; H 8.73; N 8.09. Found: C 55.68; H 8.54; N 8.15.

Nitrodioxolanes 4b,c (General procedure).

A mixture of nitroaldehyde **3b,c** (120 mmol), ethylene glycol (8 mL, 144 mmol), TsOH (190 mg, 1.1 mmol) and C_6H_6 (40 mL) was stirred under reflux with Dean-Stark apparatus until water separation finished. The reaction mixture was washed with $NaHCO_3$ saturated solution (2 × 40 mL) and H_2O (2 × 40 mL), dried with Na_2SO_4. After evaporation of the solvent under reduced pressure, the crude residue was purified via column chromatography (silica gel, $CHCl_3$) to give **4b,c**.

2-(2-(1-Nitrocyclohexyl)ethyl)-1,3-dioxolane (4b):

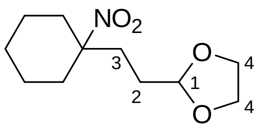

Yield : 85%, colorless liquid.

IR (neat) ν, cm^{-1}: 2939, 2868, 2763, 1535, 1450, 1427, 1412, 1348, 1230, 1141, 1036, 985, 943, 912, 864, 842.

^1H NMR (500 MHz, $CDCl_3$): 1.16-1.39 (m, 3H)(cyclohexane); 1.44-1.60 (m, 7H)(cyclohexane); 1.82-1.89 (m, 2H)($C(3)H_2$); 2.27-2.37 (m, 2H)($C(2)H_2$); 3.70-3.90 (m, 4H)(2×$C(4)H_2$); 4.75 (t, J_t=4.5, 1H)(C(1)H).

^{13}C NMR (125 MHz, $CDCl_3$): 22.1, 24.6, 27.5, 33.8, 34.0, 64.7 (2×$C(4)H_2$), 90.7 (C-NO_2), 103.2 (C(1)H).

Anal. calcd for $C_{11}H_{19}NO_4$: C 57.63; H 8.35; N 6.11. Found: C 57.36; H 8.24; N 6.08.

2-(3-Ethyl-3-nitropentyl)-1,3-dioxolane (4c):

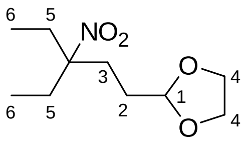

Yield : 61%, colorless liquid which solidifies when cooled to -20°C.

IR (neat) ν, cm^{-1}: 2976, 2947, 2885, 1537, 1456, 1412, 1389, 1362, 1311, 1228, 1144, 1076, 1038, 943, 914, 860, 827.

^{1}H NMR (500 MHz, $CDCl_3$): 0.79 (t, J_t=7.5, 6H)(2×C(6)H_3); 1.47-1.53 (m, 2H)(C(3)H_2); 1.88 (dq, J_d=2.7 J_q=7.5, 4H)(2×C(5)H_2); 1.94-1.99 (m, 2H)(C(2)H_2); 3.76-3.83 (m, 2H)(C(4)H_2); 3.87-3.92 (m, 2H)(C(4)H_2); 4.81 (t, J_t=4.4, 1H)(C(1)H).

^{13}C NMR (125 MHz, $CDCl_3$): 7.78 (2×C(6)H_3), 27.8, 27.9, 28.0, 64.8 (2×C(4)H_2), 94.8 (C-NO_2), 103.4 (C(1)H).

Anal. calcd for $C_{10}H_{19}NO_4$: C 55.28; H 8.82; N 6.45. Found: C 55.53; H 8.74; N 6.20.

Nitrones 5b,c (General procedure).

A solution of nitrodioxolane **4b,c** and NH_4Cl (3.1 g, 58 mmol) in H_2O/THF (2:1) mixture (60 mL) was cooled to 3 °C and Zn dust (13.0 g, 202 mmol) was added by small portions at vigorous stirring maintaining the temperature in the range of 3–6 °C. The reaction mixture was stirred for 2 hours at 10 °C, the inorganic precipitate was filtered off and washed with warm THF (60 °C, 150 mL). The combined filtrate was evaporated (temperature did not exceed 25 °C). The crude residue was dissolved in 80 mL of 5% HCl and stirred at 70 °C for 2 hours. Then the reaction mixture was cooled to 10 °C and neutralized to pH 5 with $NaHCO_3$. The resulting solution was extracted with CH_2Cl_2 (3 × 50 mL), the combined extract was washed with brine (1 × 50 mL) and dried with Na_2SO_4. Methylene chloride was evaporated and the crude residue was purified via column chromatography (silica gel, $CHCl_3$/CH_3OH).

1-Azaspiro[4.5]dec-1-ene 1-oxide (5b):

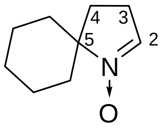

Yield : 60%, white solid, m.p 76-77 ^{o}C (Et_2O).

IR (neat) v, cm^{-1}: 2927, 2854, 1623, 1448, 1313, 1149, 114, 1070, 946, 920, 901, 848, 793.

^{1}H NMR (500 MHz, $CDCl_3$): 0.98-1.19 (m, 3H)(cyclohexane); 1.30-1.38 (m, 2H)(cyclohexane); 1.42-1.50 (m, 1H)(cyclohexane); 1.58-1.67 (m, 2H)(cyclohexane); 1.83-1.90 (m, 2H)(cyclohexane); 1.93 (t, J_t=7.6, 2H)(C(4)H_2); 2.37 (dt, J_d=2.7 J_t=7.6, 2H)(C(3)H_2); 6.60 (t, J_t=2.7, 1H)(C(2)H).

^{13}C NMR (125 MHz, $CDCl_3$): 22.6, 24.1, 24.3, 29.4, 32.8, 76.7 (C(5)), 131.4 (C(2)H).

Anal. calcd for $C_9H_{15}NO$: C 70.55; H 9.87; N 9.14. Found: C 70.87; H 9.91; N 9.31.

2,2-Diethyl-3,4-dihydro-2*H*-pyrrole 1-oxide (5c):

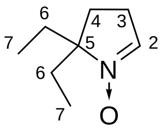

Yield : 70%, pale yellow liquid.

IR (neat) v, cm^{-1}: 2969, 2937, 2881, 1650, 1591, 1463, 1382, 1357, 1214, 1143, 1027, 1001, 930, 920, 833, 791.

^{1}H NMR (500 MHz, $CDCl_3$): 0.79 (t, J_t=7.4, 6H)(2×C(7)$\underline{H}_3$); 1.44-1.54 (m, 2H)(C(6)H_2); 1.73-1.84 (m, 2H)(C(6)H_2); 1.99 (t, J_t=7.7, 2H)(C(4)H_2); 2.41 (dt, J_d= 2.7 J_t=7.7, 2H)(C(3)H_2), 6.77 (t, J_t=2.7, 1H)(C(1)H).

^{13}C NMR (125 MHz, $CDCl_3$): 7.3 (2×C(7)H_3), 24.9, 25.8, 29.8, 79.8 (C(5)), 133.2 (C(2)H).

Anal. calcd for $C_8H_{15}NO$: C 68.04; H 10.71; N 9.92. Found: C68.43; H 10.65; N 9.78.

Pent-4-enyl nitrones 7a–c synthesis (General procedure). A solution of pent-4-enylmagnesium bromide was prepared under argon via slow addition of a solution of 5-bromopent-1-ene (1.4 g, 9.4 mmol) in dry Et_2O (15 mL) to a suspension of Mg chips (0.26 g, 11 mmol) in dry Et_2O (15 mL). Then a solution of nitrone **5a–c** (13.3 mmol) in a mixture of dry Et_2O (20 mL) and dry benzene (20 mL) was added dropwise. The reaction mixture was stirred for 3-5 h at +35°C, quenched with H_2O (5 mL) and filtered. The filtrate was evaporated under reduced pressure. The residue containing crude **6a–c** was dissolved in MeOH (50 mL) and copper (II) - ammonium complex was added (prepared via mixing of solution of $CuSO_4{\cdot}5H_2O$ (3–5 mg) in water (1 mL) with 25% aqueous NH_3 (1 mL)). Air was bubbled through the reaction mixture until conversion of starting material was complete (1 h), the progress of the reaction was monitored by TLC (silica gel, $CHCl_3$/MeOH 25:1, visualization with 1% aq $KMnO_4$). The solution was evaporated in vacuum, and the residue was dissolved in $CHCl_3$ (40 mL) and washed with a saturated solution of NaCl with the addition of 25% aqueous NH_3 (5 mL). The organic phase was evaporated under reduced pressure. The alkenyl nitrones **7a–c** were purified via column chromatography (silica gel, $CHCl_3$/MeOH 35:1).

5,5-Dimethyl-2-(pent-4-enyl)-3,4-dihydro-5*H*-pyrrole-1-oxide (7a)

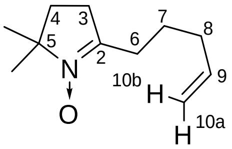

Yield : 96%, colorless oil.

UV (EtOH), λ_{max} (log ε): 237 (3.92).

IR (neat) ν, cm^{-1}: 3426, 3075, 2970, 2931, 2864, 1640, 1590, 1458, 1366, 912, 990.

^1H NMR (400 MHz, $CDCl_3$): 1.18 (s, 6H); 1.43 (quintet, J=7.7, 2H)(C(7)H_2); 1.79 (t, J_t=7.5, 2H)(C(4)H_2); 1.90 (dt, J_d=6.9Гц, J_t=7.1, 2H)(C(8)H_2); 2.28 (t, J_t=7.9, 2H)(

$C(6)H_2$); 2.38 (t, J_t=7.4, 2H)($C(3)H_2$); 4.76 (d, J_d=10, 1H)($C(10a)H_2$); 4.82 (d, J_d=17, 1H)($C(10b)H_2$); 5.59 (tdd, J_t=6.8, J_{d1}=10, J_{d2}=17) (C(9)H).

^{13}C NMR (100 MHz, $CDCl_3$): 20.09 (2×CH_3); 23.99 ($C(7)H_2$); 26.11 ($C(6)H_2$); 27.08 ($C(3)H_2$); 31.95 ($C(4)H_2$); 33.31 ($C(8)H_2$); 73.07 (C(5)); 114.97 ($C(10)H_2$); 137.40 (C(9)H); 144.16 (C(2)).

Anal. calcd for $C_{11}H_{19}NO$: C 72.88; H 10.56; N 7.73. Found: C 72.59; H 10.45; N 7.73.

2-(Pent-4-en-1-yl)-1-azaspiro[4.5]dec-1-ene 1-oxide (7b)

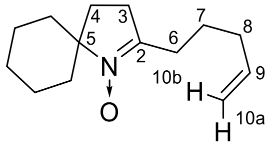

Yield : 99%, colorless oil.

UV (EtOH), λ_{max} (log ε): 235 (3.94).

IR (neat) ν, cm^{-1}: 3423, 3074, 2931, 2858, 1639, 1589, 1452, 1387, 1344, 1323, 1276, 1253, 1230, 1205, 1176, 1122, 1070, 993, 904, 852, 738, 709, 634, 567, 505.

^{1}H NMR (400 MHz, $CDCl_3$): δ =1.12-1.29 (m, 3H), 1.33-1.41 (m, 2H), 1.50-1.58 (m, 1H), 1.67-1.76 (m, 2H), 1.97-2.08 (m, 4H) (5×CH_2 spirocyclohexane and $C(8)H_2$); 1,53 (quintet, J=8, 2H)($C(7)H_2$); 1.89 (t J_t=7.5, 2H)($C(4)H_2$); 2.41 (t J_t=8, 2H)($C(6)H_2$); 2.49 (t J_t=7.2, 2H)($C(3)H_2$); 4.87 (ddt J_{d1}=10 J_{d2}=1.9 J_t=1.2, 1H)(C(10a)H); 4.93 (ddt J_{d1}=17 J_{d2}=1.9 J_t=1.5, 1H)(C(10b)H); 5.70 (ddt J_{d1}=17 J_{d2}=10 J_t=6.8, 1H)(C(9)H).

^{13}C NMR (100 MHz, $CDCl_3$): 22.58, 23.46, 25.26, 25.93, 29.65, 32.49, 33.33, 33.58, 38.33, 41.34 ($C(3)H_2$, $C(4)H_2$, $C(6)H_2$, $C(7)H_2$, $C(8)H_2$ and 5×CH_2 spirocyclohexane), 58.21 (C(9)H), 70.48 (C(5)), 71.49 ($C(10)H_2$-O), 82.37 (C(2)-N).

Anal. calcd for $C_{14}H_{23}NO$: C 75.97; H 10.47; N 6.33. Found: C 75.90; H 10.43; N 6.29.

5,5-Diethyl-2-(pent-4-enyl)-3,4-dihydro-5*H*-pyrrole-1-oxide (7c)

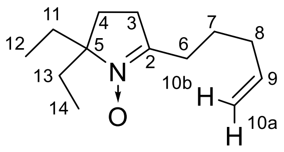

Yield : 94%, colorless oil.

UV (EtOH), λ_{max} (log ε): 235 (3.94).

IR (neat) ν, cm^{-1}: 3076, 2970, 2927, 2881, 2858, 1641, 1598, 1459, 1440, 1378, 1276, 1220, 1145, 1066, 1037, 995, 910, 790, 738, 707, 630, 570.

^{1}H NMR (500 MHz, $CDCl_3$): 0.73 (t, J_t=7.4, 6H)(C(12)H_2 and C(14)H_2); 1.47 (qd Jq=7.4 Jd=13.9, 2H)(one from each group C(11)H_2 and C(13)H_2); 1.53 (tt J_{t1}=7.5 J_{t2}=7.7, 2H)(C(7)H_2); 1.81 (qd J_q=7.4 $J_{\text{д}}$=13.9, 2H)(one from each group C(11)H_2 and C(13)H_2); 1.85 (t J_t=7.9, 2H)(C(4)H_2); 2.01 (dddt J_{d1}=1.2 J_{d2}=1.6 J_{d3}=6.7 J_t=7.5, 2H)(C(8)H_2); 2.40 (tt J_{t1}=1.3 J_{t2}=7.9, 2H)(C(3)H_2); 2.44 (tt J_{t1}=1.28 J_{t2}=7.7, 2H)(C(6)H_2); 4.87 (ddt J_{d1}=1.9 J_{d2}=10.2 J_t=1.2, 1H)(C(10a)H); 4.92 (ddt J_{d1}=1.9 J_{d2}=17.1 J_t=1.6, 1H)(C(10b)H); 5.7 (ddt J_{d1}=10.2 J_{d2}=17.1 J_t=6.7, 1H)(C(9)H).

^{13}C NMR (125 MHz, $CDCl_3$): 7.69 (C(12)H_3 and C(14)H_3), 24.17, 24.34, 26.19, 28.45, 30.29 (C(11)H_2 and C(13)H_2), 33.65, 79.96 (C(5)), 115.20 (C(10)H_2=), 137.68 (C(9)H=), 146.03 (C(2)=N).

Anal. calcd for $C_{13}H_{23}NO$: C 74.59; H 11.08; N 6.69. Found: C 74.37; H 11.07; N 6.32.

Intramolecular 1,3-dipolar cycloaddition of nitrones (General procedure). TEMPO (2–3 mg) was added to a solution of alkenyl nitrone **7a–c** (7.2 mmol) in toluene (20 mL) and the mixture was bubbled with Ar, and then it was heated under microwave irradiation at 145 °C for 30–60 minutes. After evaporation of the solvent, the crude residue was purified via column chromatography (silica gel, Et_2O/C_6H_{14} 4:3) to give **8a–c**.

3,3-Dimethylhexahydro-1*H*,6*H*-cyclopenta[*c*]pyrrolo[1,2-*b*]isoxazole (8a).

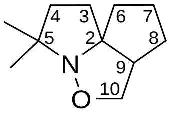

Yield : 92%. colorless oil.

IR (neat) ν, cm^{-1}: 3382, 2953, 2866, 1465, 1380, 1364, 1310, 1251.

^1H NMR (400 MHz, $CDCl_3$): δ = 1.05 (s, 3H); 1.44 (s, 3H); 1.44-1.56 (m, 3H); 1.56-1.66 (m, 2H); 1.69-1.77 (m, 1H); 1.82-1.95 (m, 3H); 1.99-2.06 (m, 1H); 2.52-2.59 (m, 1H, C(9)H); 3.25 (t, J=8.5, 1H)(C(10)H_2); 3.95 (t, J=8.5, 1H)(C(10)H_2).

^{13}C NMR (100 MHz, $CDCl_3$): δ = 24.24, 25.73 (2×CH_3); 25.22 (C(7)H_2); 29.61 (C(8)H_2); 36.42 (C(3)H_2); 39.02 (C(4)H_2); 41.54 (C(6)H_2); 58.28 (C(9)H); 67.24 (C(5)); 71.41 (C(10)H_2); 83.24 (C(2)).

Anal. calcd for $C_{11}H_{19}NO$: C 72.88; H 10.56; N 7.73; Found: C 72.68; H 10.49; N 7.80.

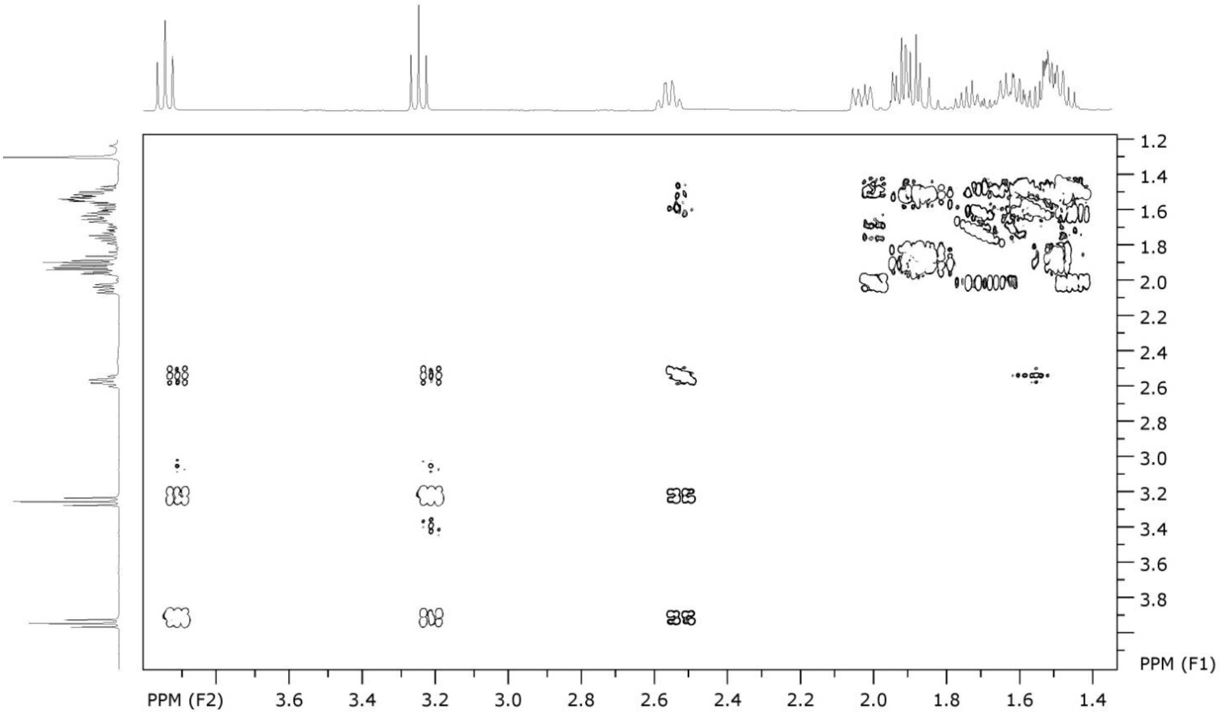

Figure S1: COSY $^1H,^1H$ of cycloadduct **8a.**

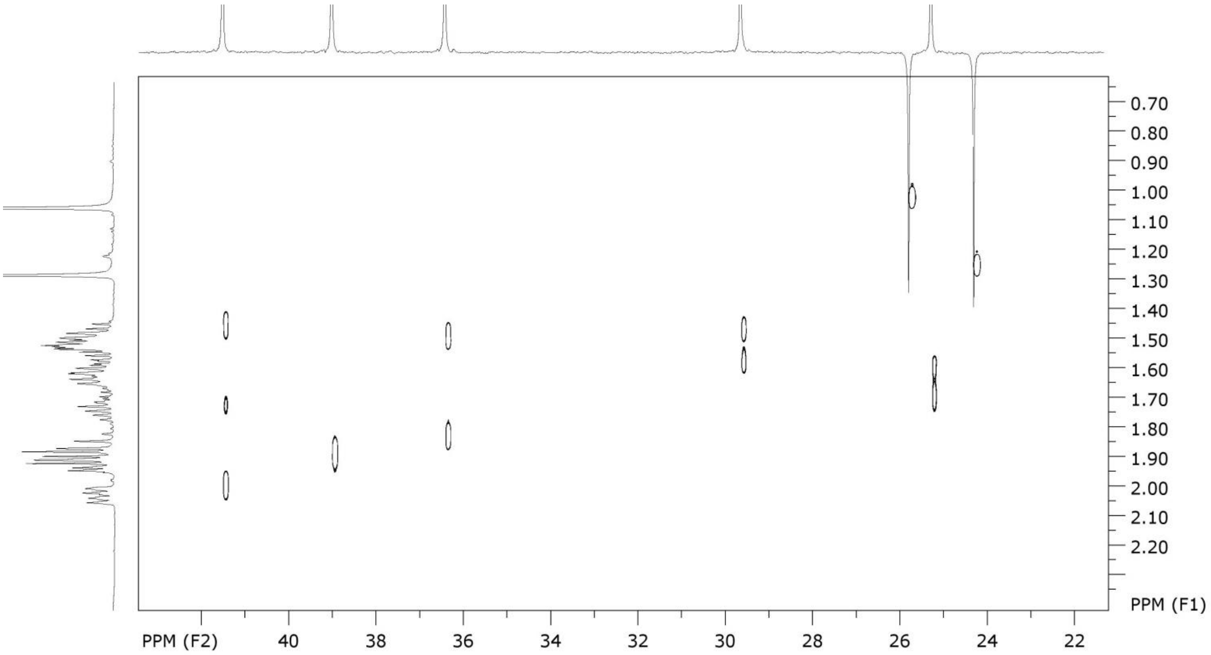

Figure S2: HSQC $^{1}H,^{13}C$ of cycloadduct **8a.**

Hexahydro-6'*H*-spiro[cyclohexane-1,3'-cyclopenta[*c*]pyrrolo[1,2-*b*]isoxazole] (8b).

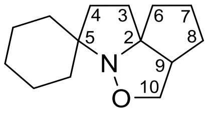

Yield: 98%, colorless oil.

IR (neat) ν, cm^{-1}: 2935, 2856, 1448, 1351, 1305, 1296, 1259, 1228, 1174, 1151, 1108, 1054, 1041, 987, 975, 918, 910, 854, 802, 769, 626.

^{1}H NMR (500 MHz, $CDCl_3$): δ = 1.17-1.30 (m, 5H); 1.38-1.45 (m, 2H); 1.45-1.51 (m, 2H); 1.54-1.63 (m, 3H); 1.63-1.69 (m, 3H); 1.69-1.77 (m, 3H); 1.79-1.87 (m, 1H); 1.87-1.94 (m, 1H); 1.95-2.01 (m, 1H), 2.49-2.56 (m, 1H)(C(9)H), 3.23 (t J_t=8.5, 1H)(C(10)H_2); 3.90 (t J_t=8.5, 1H)(C(10)H_2).

^{13}C NMR (125 MHz, $CDCl_3$): 22.58, 23.46, 25.26, 25.93, 29.65, 32.49, 33.33, 33.58, 38.33, 41.34 (C(3)H_2, C(4)H_2, C(6)H_2, C(7)H_2, C(8)H_2 and 5×CH_2 spirocyclohexane), 58.21 (C(9)H), 70.48 (C(5)), 71.49 (C(10)H_2-O), 82.37 (C(2)-N).

Anal. calcd for $C_{14}H_{23}NO$: C 75.97; H 10.47; N 6.33. Found: C 76.09; H 10.52; N 6.17.

3,3-Diethylhexahydro-1*H*,6*H*-cyclopenta[*c*]pyrrolo[1,2-*b*]isoxazole (8c).

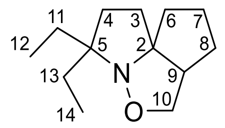

Yield : 63%, colorless oil.

IR (neat) ν, cm^{-1}: 2958, 2875, 2865, 1461, 1377, 1353, 1303, 1296, 1286, 1174, 1139, 1054, 1043, 972, 950, 916, 856, 813, 790, 759, 626, 576, 559, 524, 495.

^{1}H NMR (500 MHz, $CDCl_3$): 0.84 (t, J_t=7.5, 3H) and 0.90 (t, J_t=7.5, 3H)(C(12)H_3 and C(12)H_3); 1.24-1.33 (m, 1H); 1.41-1.54 (m, 3H); 1.55-1.60 (m, 1H); 1.60-1.70 (m, 4H); 1.72-1.80 (m, 2H); 1.80-1.86 (m, 1H); 1.89-1.94 (m, 1H); 1.99-2.05 (m, 1H); 2.50-2.56 (m, 1H)(C(9)H); 3.29 (t, J_t=8, 1H)(C(10)H_2); 3.91 (t, J_t=8, 1H)(C(10)H_2).

^{13}C NMR (125 MHz, $CDCl_3$): 8.25 and 9.29 (C(12)H_3 and C(14)H_3), 25.46, 25.53, 25.79, 30.14, 34.09, 38.70, 41.72 (C(3)H_2, C(4)H_2, C(6)H_2, C(7)H_2, C(8)H_2, C(11)H_2, C(13)H_2), 58.54 (C(9)H), 72.08 (C(10)H_2), 73.92 (C(5)), 83.36 (C(2)).

Anal. calcd for $C_{13}H_{23}NO$: C 74.59; H 11.08; N 6.69. Found: C 74.39; H 11.16; N 6.70.

General procedure for isoxazolidine ring opening (9a–c synthesis). Zn powder (3.11 g, 47.8 mmol) was added in one portion to a warm (60 ^{o}C) stirred solution containing isoxazolidine **8a–c** (4.78 mmol), EtOH (9 mL), 10 M AcOH (30 mL) and EDTA disodium salt (8 g). The reaction mixture was stirred at 60 ^{o}C for 3 h and then cooled down to rt. The mixture was basified to pH 10 with 25% NH_3 solution and

extracted with EtOAc. The organic extract was dried with Na_2CO_3. After evaporation of the solvent, the crude residue was purified via column chromatography (silica gel EtOAc/MeOH 5:1) to give aminoalcohols **9a–c**.

(2,2-Dimethyl-1-azaspiro[4.4]nonan-6-yl)methanol (9a).

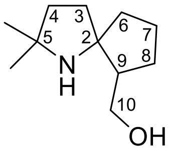

Yield : 90%, colorless oil.

IR (neat) ν, cm^{-1}: 3289, 2954, 2868, 1460, 1379, 1364.

^1H NMR (600 MHz, $CDCl_3$): 1.15 (s, 3H); 1.19 (s, 3H); 1.29-1.39 (m, 1H); 1.48-1.64 (m, 5H); 1.66-1.86 (m, 5H); 3.58 (d, J_d=5.7)(C(10)H_2).

^{13}C NMR (150 MHz, $CDCl_3$): 29.56, 29.99 (2×CH_3), 21.43 (C(7)H_2), 26.06 (C(8)H_2), 38.54 (C(3)H_2), 38.91 (C(6)H_2), 39.92 (C(4)H_2), 47.87 (C(9)H), 64.83 (C(10)H_2), 59.26 (C(5)), 72.98 (C(2)).

Anal. calcd for $C_{11}H_{21}NO$: C 72.08; H 11.55; N 7.64. Found: C 72.26; H 8.12; N 11.92.

(6-Azadispiro[4.1.5^7.2^5]tetradecan-1-yl)methanol (9b).

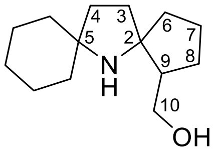

Yield : 99%, colorless crystals; m.p. 37.8-39.6 (EtOAc/MeOH).

IR (KBr) ν, cm^{-1}: 3247, 3105, 2931, 2854, 1446, 1338, 1321, 1255, 1191, 1176, 1134, 1107, 1093, 1037, 997, 937, 914, 891, 848, 808, 783, 761.

^1H NMR (600 MHz, $CDCl_3$): 1.22-1.41 (m, 7H); 1.41-1.52 (m, 6H); 1.52-1.61 (m, 3H); 1.63-1.71 (m, 4H); 1.71-1.77 (m, 1H); 3.55 (d J_d=5.4, 2H)(C(10)H_2).

^{13}C NMR (150 MHz, $CDCl_3$): 21.49, 23.48, 23.74, 25.51, 26.00, 35.76, 37.46, 38.69, 39.50, 40.21(C(3)H_2, C(4)H_2, C(6)H_2, C(7)H_2, C(8)H_2 and 5×CH_2 spirocyclohexane), 47.47 (C(9)H), 61.64 (C(5)), 64.58 (C(10)H_2-O), 71.95 (C(2)-N).

Anal. calcd for $C_{14}H_{25}NO$: C 75.28; H 11.28; N 6.07. Found: C 75.75; H 10.94; N 6.22.

(2,2-Diethyl-1-azaspiro[4.4]nonan-6-yl)methanol (9c).

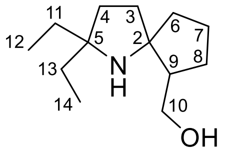

Yield : 85%, colorless oil.

IR (neat) ν, cm^{-1} : 3295, 2958, 2875, 1458, 1377, 1336, 1299, 1213, 1188, 1170, 1141, 1097, 1081, 1064, 1039, 979, 950, 916, 877, 831, 796, 736, 673, 663, 553.

^{1}H NMR (300 MHz, $CDCl_3$): 0.78 (t, J_t=7.5, 3H) and 0.80 (t, J_t=7.5, 3H)(C(12)H_3 and C(14)H_3); 1.26-1.36 (m, 1H); 1.36-1.48 (m, 4H); 1.51-1.66 (m, 6H); 1.67-1.80 (m, 4H); 3.57 (d, J_d=5.7, 2H)(C(10)H_2).

^{13}C NMR (125 MHz, $CDCl_3$): 8.90 and 9.10 (C(12)H_3 and C(14)H_3), 21.81, 26.34, 31.19, 31.35, 35.27, 38.06, 40.32, 47.84 (C(9)H), 65.03 (C(10)H_2), 65.12 (C(5)), 72.61 (C(2)).

Anal. calcd for $C_{13}H_{25}NO$: C 73.88; H 11.92; N 6.63. Found: C 73.16; H 11.97; N 6.49.

Acetylation of aminoalcohols 9a–c, 19 (General procedure). Acetic anhydride (0.56 g; 5.5 mmol or 1.12 g; 11 mmol for **19**) was added to a solution of aminoalcohol **9a–c, 19** (3.1 mmol) in dry chloroform (15 mL) and the mixture was refluxed until the reaction was complete. The progress of the reaction was monitored by TLC (silica gel, C_6H_{14}/EtOAc 5:1, visualization with 1% aq $KMnO_4$). The reaction mixture was washed with a saturated solution of Na_2CO_3 and dried with Na_2SO_4. After evaporation of the

solvent, the crude residue was purified via column chromatography (silica gel, C_6H_{14}/EtOAc 5:1) to give **10a–c**, **20**.

(2,2-Dimethyl-1-azaspiro[4.4]nonan-6-yl)methyl acetate (10a).

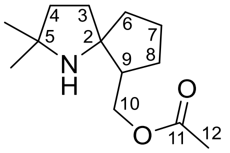

Yield: 92%, colorless oil.

IR (neat) ν, cm^{-1}: 3454, 3350, 2956, 2867, 1737, 1459, 1377, 1363, 1241, 1192, 1162, 1031, 971.

^{1}H NMR (400 MHz, $CDCl_3$): 1.08 (s, 3H); 1.15 (s, 3H); 1.36-1.44 (m, 1H); 1.47-1.54 (m, 1H); 1.55-1.63 (m, 5H), 1.70-1.79 (m, 2H); 1.80-1.89 (m, 2H); 2.01 (s. 3H)(C(12)H_3); 3.97 (dd J_{d1}=11 J_{d2}=7.6, 1H)(C(10)H_2); 4.25 (dd J_{d1}=11 J_{d2}=6, 1H)(C(10)H_2).

^{13}C NMR (125 MHz, $CDCl_3$): 21.16, 30.64 and 30.81 (3×CH_3), 21.24, 27.88, 38.32, 39.55, 41.87 (C(3)H_2, C(4)H_2, C(6)H_2, C(7)H_2, C(8)H_2), 47.07 (C(9)H), 59.05 (C(5)), 66.34 (C(10)H_2O), 71.13 (C(2)), 171.25 (C(11)=O).

Anal. calcd for $C_{13}H_{23}NO_2$: C 69.29; H 10.29; N 6.22. Found: C 69.45; H 10.35; N 6.35.

(6-Azadispiro[4.1.5^7.2^5]tetradecan-1-yl) methyl acetate (10b).

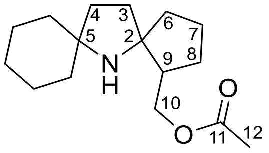

Yield : 87%, colorless oil.

IR (neat) ν, cm^{-1}: 3454, 3349, 2927, 2856, 1737, 1448, 1384, 1365, 1238, 1172, 1135, 1087, 1031, 972.

^{1}H NMR (500 MHz, $CDCl_3$): 1.20-1.44 (m, 8H); 1.44-1.63 (m, 9H); 1.63-1.68 (m, 1H); 1.71-1.79 (m, 2H); 1.80-1.88 (m, 1H); 2.01 (s, 3H)(C(12)H_3); 3.96 (dd J_{d1}=11 J_{d2}=7.9, 1H)(C(10)H_2); 4.26 (dd J_{d1}=11 J_{d2}=5.7, 1H)(C(10)H_2).

^{13}C NMR (125 MHz, $CDCl_3$): 21.03 (C(12)H_3), 21.18, 23.77, 23.97, 25.70, 27.83, 36.09, 37.39, 39.62, 40.19, 41.78 (C(3)H_2, C(4)H_2, C(6)H_2, C(7)H_2, C(8)H_2 and 5×CH_2 spirocyclohexane), 46.67 (C(9)H), 61.38 (C(5)), 66.42 (C(10)H_2O), 70.00 (C(2)-N), 171.20 (C(11)=O).

Anal. calcd for $C_{16}H_{27}NO_2$: C 72.41; H 10.25; N 5.28. Found: C 72.66; H 10.26; N 5.27.

(2,2-Diethyl-1-azaspiro[4.4]nonan-6-yl)methyl acetate (10c).

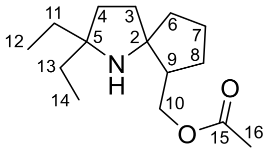

Yield: 71%, colorless oil.

IR (neat) ν, cm^{-1}: 3457, 3357, 2958, 2875, 1739, 1458, 1419, 1382, 1365, 1313, 1240, 1180, 1157, 1145, 1074, 1031, 973.

^{1}H NMR (300 MHz, $CDCl_3$): 0.77 (t, J_t=7.5, 6H)(C(12)H_3 and C(14)H_3); 1.25-1.45 (m, 5H); 1.45-1.69 (m, 7H); 1.69-1.88 (3H); 1.99 (c, 3H)(C(16)H_3); 3.96 (dd, J_{d1}=7.8 J_{d2}=10.9, 1H)(C(10)H_2); 4.26 (dd, J_{d1}=5.7 J_{d2}=10.9, 1H)(C(10)H_2).

^{13}C NMR (75 MHz, $CDCl_3$): 8.23 and 9.25 (C(12)H_3 and C(14)H_3), 21.23 (C(16)H_3), 21.48, 28.08, 31.78, 32.11, 35.62, 37.87, 41.94, 47.10 (C(9)H), 64.52 (C(5)), 66.71 (C(10)H_2), 70.50 (C(2)), 171.41 (C(15)=O).

Anal. calcd for $C_{15}H_{27}NO_2$: C 71.10; H 10.74; N 5.53. Found: C 71.46; H 10.74; N 5.70.

((1*R*,5*S*,7*S*,8*R*,12*S*,13*S*)-12,13-di-*tert*-butoxy-6-azadispiro[4.1.4^{7}.2^{5}]tridecane-1,8-diyl)bis(methylene) diacetate (20).

Yield: 90 %, colorless oil.

^{1}H NMR (500 MHz, $CDCl_3$): 1.20 (s, 18H); 1.34-1.41 (m, 4H); 1.44-1.51 (m, 2H); 1.57-1.64 (m, 2H); 1.66-1.73 (2H); 1.95-2.02 (m, 2H); 2.03 (s, 3H); 2.04 (s, 3H); 2.24-2.31 (m, 2H); 3.77 (s, 2H); 4.03 (dd, J_1=6.7, J_2=11.3, 2H); 4.24 (dd, J_1=6.3, J_2=11.3, 2H); 6.44 (br s, 1H).

^{13}C NMR (125 MHz, $CDCl_3$): 20.10 (cyclopentane), 20.90 (CH_3), 27.12 (cyclopentane), 29.29 (CH_3, ^{t}Bu), 34.78 (cyclopentane), 42.21 (CH, cyclopentane), 65.10 (CH_2O), 69.02 (C, ^{t}Bu), 73.49 (C-N), 78.61 (CH-O), 170.99 (C=O).

Anal. calcd for $C_{26}H_{45}NO_6$: C 66.78; H 9.70; N 3.00. Found: C 66.58; H 9.56; N 3.17.

Nitroxides 11a–c, 17, 21 synthesis (General procedure). A solution of amine **10a–c, 20** (0.38 mmol) in $CHCl_3$ (4 mL) was cooled to –10 °C and *m*-CPBA (98 mg, 0.57 mmol or 196 mg, 1.14 mmol for **20**) was added portionwise upon stirring. The mixture was stirred at 0 °C until the reaction was complete (control by TLC, silica gel Et_2O/hexane 1:1). After evaporation of the solvent, the crude residue was purified via column chromatography (silica gel, Et_2O/C_6H_{14} 1:1).

6-(Acetoxymethyl)-2,2-dimethyl-1-azaspiro[4.4]nonan 1-oxyl (11a).

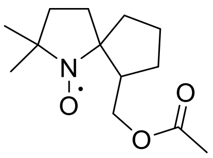

Yield: 70%, yellow oil.

UV (EtOH), λ_{max} (log ε): 240 (3.28).

IR (neat) ν, cm^{-1}: 2968, 2873, 1741, 1461, 1434, 1388, 1365, 1321, 1238, 1128, 1033, 752, 700, 644, 605.

Anal. calcd for $C_{13}H_{22}NO_3$: C 64.97; H 9.23; N 5.83. Found: C 64.75; H 9.29; N 5.99.

1-(Acetoxymethyl)-6-azadispiro[4.1.5^7.2^5]tetradecan 6-oxyl (11b).

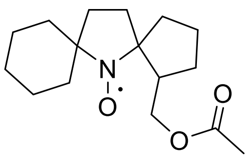

Yield: 99%, yellow oil.

UV (EtOH), λ_{max} (log ε): 232 (3.41).

IR (neat) ν, cm^{-1}: 2935, 2858, 1739, 1450, 1388, 1365, 1236, 1184, 1033, 914, 605.

Anal. calcd for $C_{16}H_{26}NO_3$: C 68.54; H 9.35; N 5.00. Found: C 68.79; H 9.37; N 4.83.

6-(Acetoxymethyl)-2,2-diethyl-1-azaspiro[4.4]nonan 1-oxyl (11c).

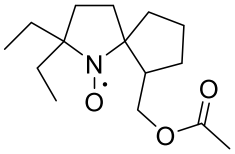

Yield: 82%, yellow oil.

UV (EtOH), λ_{max} (log ε): 240 (3.23).

IR (neat) ν, cm^{-1}: 2964, 2879, 1741, 1461, 1404, 1384, 1365, 1274, 1238, 1182, 1122, 1108, 1033, 973, 923, 873, 646, 605, 555.

Anal. calcd for $C_{15}H_{26}NO_3$: C 67.13; H 9.77; N 5.22. Found: C 67.21; H 9.47; N 5.51.

9-(Acetoxymethyl)-2,2-dimethyl-1-azaspiro[4.4]non-6-en 1-oxyl (17).

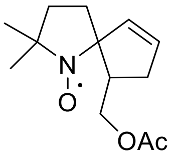

Yield: 22%, yellow oil.

UV (EtOH), λ_{max} (log ε): 228 (3.40).

IR (neat) ν, cm^{-1}: 3054, 2970, 2933, 2875, 1741, 1620, 1573, 1539, 1459, 1386, 1365, 1324, 1234, 1134, 1080, 1070, 1031, 973, 962, 896, 856, 835, 744, 698, 669, 646, 605, 582.

(1*R*,5*S*,7*S*,8*R*,12*S*,13*S*)-1,8-bis(acetoxymethyl)-12,13-di-*tert*-butoxy-6-azadispiro[4.1.4^7.2^5]tridecan 6-oxyl (21).

Yield: 75 %, yellow oil.

UV (EtOH), λ_{max} (log ε): 229 (3.39).

IR (neat) ν, cm^{-1}: 2972, 2959, 2871, 1736, 1470, 1393, ,1366, 1256, 1192, 1124, 1076, 1028, 997, 930, 910,750, 719.

Anal. calcd for $C_{26}H_{44}NO_7$: C 64.70; H 9.19; N 2.90. Found: C 64.80; H 9.26; N 3.11.

Synthesis of nitroxides 1, 12a–c (General procedure). Aqueous NH_3 (15 mL) was added to a solution of nitroxide **11a–c**, **21** (3.6 mmol) in MeOH (30 mL), and allowed to stand at +20 to +40 ^oC until the reaction was complete (up to 12 hours). The progress of the reaction was monitored by TLC (silica gel, Et_2O/C_6H_{14} 2:1, visualization with 1% aq $KMnO_4$). The solution was evaporated under reduced pressure. The residue was dissolved in the saturated solution of NaCl and extracted with $CHCl_3$ (3 × 10 mL). The extract was dried with Na_2SO_4. After evaporation of the solvent, the crude residue was purified via column chromatography (silica gel, Et_2O/C_6H_{14} 2:1).

6-(Hydroxymethyl)-2,2-dimethyl-1-azaspiro[4.4]nonan 1-oxyl (12a).

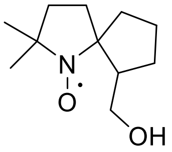

Yield: 85%, yellow crystals, m.p. 41.5-43 ^oC (hexane).

UV (EtOH), λ_{max} (log ε): 246 (3.16).

IR (KBr) ν, cm^{-1}: 3319, 2966, 2931, 2867, 1461, 1409, 1370, 1359, 1319, 1249, 1236, 1182, 1098, 1034, 1022.

Anal. calcd for $C_{11}H_{20}NO_2$: C 66.63: H 10.17: N 7.06: Found: C 66.45: H 10.01: N 7.10.

1-(Hydroxymethyl)- 6-azadispiro[4.1.5^7.2^5]tetradecan 6-oxyl (12b).

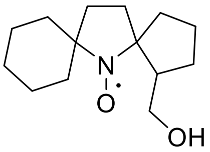

Yield : 88%, yellow crystals, m.p. 38.9-39.4 ^{o}C (hexane/Et_2O).

UV (EtOH), λ_{max} (log ε): 238 (3.29).

IR (KBr) ν, cm^{-1}: 3301, 2933, 2858, 1635, 1452, 1404, 1326, 1311, 1242, 1170, 1085, 1039.

Anal. calcd for $C_{14}H_{24}NO_2$: C 70.55; H 10.15; N 5.88. Found: C 70.38; H 10.12; N 5.86.

6-(Hydroxymethyl)-2,2-dimethyl-1-azaspiro[4.4]nonan 1-oxyl (12c).

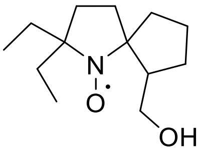

Yield : 94%, yellow oil.

UV (EtOH), λ_{max} (log ε): 240 (3.26).

IR (neat) ν, cm^{-1}: 3421, 2964, 2877, 1461, 1404, 1380, 1326, 1307, 1242, 1232, 1197, 1168, 1087, 1033, 1004.

Anal. calcd for $C_{13}H_{24}NO_2$: C 68.99; H 10.69; N 6.09. Found: C 69.22; H 10.42; N 6.09.

6-Formyl-2,2-dimethyl-1-azaspiro[4.4]nonan 1-oxyl (15). A solution of **9a** (90 mg, 0.5 mmol) in dry $CHCl_3$ (4 mL) was cooled to −10 °C and *m*-CPBA (140 mg, 0.75 mmol)

was added in one portion. The solution was stirred at 0 °C for 3 hours, then one more portion of *m*-CPBA (280 mg, 1.5 mmol) was added. The solution was stirred at 0 °C for 2 hours and 10 hours at room temperature. The reaction mixture was washed with saturated Na_2CO_3, the organic layer dried with Na_2CO_3. After evaporation of the solvent, the crude residue was purified via short flash column chromatography (silica gel, EtOAc).

Yield : 73%, yellow oil.

UV (EtOH), λ_{max} (log ε): 230 (3.37).

IR (neat) ν, cm^{-1}: 2966, 1725, 1657, 1630, 1538, 1457, 1437, 1386, 1256.

Anal. calcd for $C_{11}H_{18}NO_2$: C 67.32; H 9.24; N 7.14. Found: C 67.69; H 9.45; N 7.50.

Reduction of 15 by $NaBH_4$

A solution of **15** (50 mg, 0.26 mmol) in EtOH (5 mL) was cooled to 0 °C and $NaBH_4$ (20 mg, 0.53 mmol) was added in one portion. The suspension was stirred for 3 hours at room temperature. The reaction mixture was diluted with H_2O (10 mL) and extracted with $CHCl_3$ (3 × 5 mL). The extract was dried with Na_2SO_4. After evaporation of the solvent, the crude residue was purified via column chromatography (silica gel, Et_2O/C_6H_{14} 2:1) to give **12a** with an almost quantitative yield (49 mg, 97%).

Alkoxyamines 16a–c synthesis (General procedure).

Alkoxyamines were prepared using the method developed by Matyjaszewski [5]. Nitroxide **11a–c** (2.02 mmol), dissolved in dry benzene (15 mL), benzyl bromide (0.449 g; 2.63 mmol), copper (II) triflate (0.033 g; 0.1 mmol), 4,4'-di-*tert*-butyl-2,2'-bipyridyl (0.027 g; 0.1 mmol), Cu (fine powder; 1.9 g; 30.3 mmol) were placed in a Schlenk flask and degassed by three times repeated freeze-pump-thaw procedure. The mixture was stirred for 48 hours at 60 °C. The precipitate was filtered off, the filtrate was washed

with a saturated ammonia solution (3 × 15 mL) and dried with Na_2SO_4. After evaporation of the solvent, the crude residue was purified via column chromatography (silica gel, Et_2O/C_6H_{14} 1:5).

(1-(Benzyloxy)-2,2-dimethyl-1-azaspiro[4.4]nonan-6-yl)methyl acetate (16a).

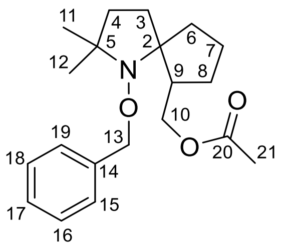

Yield: 74%, colorless oil.

IR (neat) ν, cm^{-1}: 3089, 3064, 3031, 2958, 2871, 1737, 1610, 1496, 1451, 1363, 1317, 1238, 1191, 1172, 1139, 1081, 1029, 975, 946, 908, 842, 752, 734, 696, 607, 530.

^{1}H NMR (400 MHz, $CDCl_3$): 1.15 and 1.24 (s both, 2×3H)(C(11)H_3 and C(12)H_3); 1.38-1.56 (m, 5H); 1.67-1.75 (m, 1H); 1.79-1.88 (m, 3H); 1.93-2.02 (m, 1H); 2.05 (s, 3H)($^{21}CH_3$); 2.43-2.55 (m, 1H)(C(9)H); 4.21 (dd, J_{d1}=8.4 J_{d2}=11.0, 1H)(C(10)H_2); 4.47 (dd, $J_{д1}$=5.5 J_{d2}=11.0, 1H)(C(10)H_2); 4.72 (d, J=10.8, 1H)(C(13)H_2); 4.77 (d, J=10.8, 1H)(C(13)H_2); 7.25-7.35 (m, 5H)(CH_{ar}).

^{13}C NMR (100 MHz, $CDCl_3$): 21.30 and 22.40 (C(11)H_3 and C(12)H_3), 30.24 (C(21)H_3), 23.23, 29.74, 35.08, 35.58, 35.92 (C(3)H_2, C(4)H_2, C(6)H_2, C(7)H_2, C(8)H_2), 49.59 (C(9)H), 65.50 (C(5)), 66.51 (C(10)H_2), 75.17 (C(2)), 76.62 (C(13)H_2), 127.61 (C(17)H), 128.04 (C(15)H and C(19)H), 128.38 (C(16)H and C(18)H), 137.92 (C(14)), 171.53 (C(20)=O).

Anal. calcd for $C_{20}H_{29}NO_3$: C 72.47; H 8.82; N 4.23. Found: C 72.19; H 8.40; N 4.00.

(6-(Benzyloxy)-6-azadispiro[4.1.5^7.2^5]tetradecan-1-yl)methyl acetate (16b).

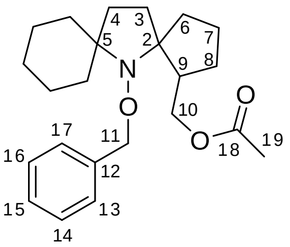

Yield : 95%, colorless oil.

IR (neat) ν, cm^{-1}: 3089, 3064, 3031, 2933, 2858, 1737, 1608, 1496, 1450, 1386, 1365, 1324, 1309, 1236, 1180, 1157, 1105, 1080, 1029, 970, 918, 842, 752, 734, 696, 648, 605, 551, 513, 462.

^{1}H NMR (500 MHz, $CDCl_3$): 1.10 (tdd, J_T=3.6 J_{d1}= J_{d2}=12.9, 1H); 1.21-1.33 (m, 3H); 1.40-1.54 (m, 4H); 1.54-1.62 (m, 3H); 1.62-1.76 (m, 6H); 1.76-1.79 (m, 1H); 1.79-1.87 (m, 2H); 1.94-2.01 (m, 1H); 2.04 (s, 3H)(C(19)H_3); 2.40-2.52 (m, 1H)(C(9)H); 4.20 (dd, J_{d1}=8.7 J_{d2}=11.0, 1H)(C(10)H_2); 4.47 (dd, J_{d1}=5.3 J_{d2}=11.0, 1H)(C(10)H_2); 4.76 (d, J_d=10.9)(C(11)H_2); 4.79 (d, J_d=10.9, 1H)(C(11)H_2); 7.23-7.28 (m, 1H)(CH_{ar}); 7.30-7.33 (m, 4H)(CH_{ar}).

^{13}C NMR (125 MHz, $CDCl_3$): 21.25 (C(19)H_3), 23.44, 23.86, 24.93, 26.14, 30.02, 31.03, 31.80, 35.04, 35.35, 39.49 (C(3)H_2, C(4)H_2, C(6)H_2, C(7)H_2, C(8)H_2 and 5×CH_2 cyclohexane), 49.07 (C(9)H), 66.56 (C(10)H_2), 69.11 (C(5)), 75.06 (C(2)), 76.72 (C(11)H_2), 127.55 (C(15)H), 127.93 and 128.38 (C(17)H, C(21)H and C(18)H, C(20)H), 138.02 (C(12)), 171.42 (C(18)=O).

Anal. calcd for $C_{23}H_{33}NO_3$: C 74.36; H 8.95; N 3.77. Found: C 75.77; H 9.35; N 3.86.

(1-(Benzyloxy)-2,2-diethyl-1-azaspiro[4.4]nonan-6-yl)methyl acetate (16c).

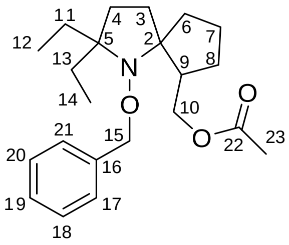

Yield: 70%, colorless oil.

IR (neat) ν, cm^{-1}: 3108, 3089, 3064, 3031, 2960, 2877, 1739, 1608, 1587, 1496, 1461, 1452, 1382, 1365, 1307, 1236, 1102, 1155, 1122, 1080, 1029, 973, 927, 910, 883, 846, 783, 752, 734, 696, 669, 605, 489, 464.

^{1}H NMR (500 MHz, $CDCl_3$): 0.86 (t, J_t=7.6, 3H) and 0.99 (t, J_t=7.4, 3H)(C(12)H_3 and C(14)H_3); 1.39-1.54 (m, 5H); 1.54-1.64 (m, 3H); 1.64-1.74 (m, 2H); 1.74-1.84 (m, 3H); 1.96-2.02 (m, 1H); 2.03 (s, 3H)(C(23)H_3); 2.43-2.53 (m, 1H)(C(9)H); 4.23 (dd, J_{d1}=8.6 J_{d2}=10.8, 1H)(C(10)H_2); 4.51 (dd J_{d1}=5.5 J_{d2}=10.8, 1H)(C(10)H_2); 4.70 (d, J_d=10.6, 1H)(C(15)H_2); 4.74 (d, J_d=10.6, 1H)(C(15)H_2); 7.22-7.27 (m, 1H)(CH_{ar}); 7.27-7.33 (m, 4H)(CH_{ar}).

^{13}C NMR (125 MHz, $CDCl_3$): 8.78 and 9.57 (C(12)H_3 and C(14)H_3), 21.24 (C(23)H_3), 23.31, 28.10, 29.95, 30.38, 30.63 34.77, 35.53 (C(3)H_2, C(4)H_2, C(6)H_2, C(7)H_2, C(8)H_2, C(11)H_2, C(13)H_2), 49.02 (C(9)H), 66.53 (C(10)H_2), 70.88 (C(5)), 75.67 (C(2)), 75.83 (C(15)H_2), 127.57 (C(19)H), 128.01 and 128.37 (C(17)H, C(21)H and C(18)H, C(20)H), 137.92 (C(16)), 171.39 (C(22)=O).

Anal. calcd for $C_{22}H_{33}NO_3$: C 73.50; H 9.25; N 3.90. Found: C 73.64; H 9.38; N 4.13.

(1-Methoxy-2,2-dimethyl-1-azaspiro[4.4]non-8-en-6-yl)methyl acetate (18). In analogy to literature method[6], the mixture of **17** (0.3 g; 1.26 mmol), acetone (2 mL) and 30% aqueous H_2O_2 (0.9 mL, 8.44 mmol) was cooled to 0°C in the ice bath and

CuCl (9 mg, 0.0088 mmol) was added upon vigorous stirring. The reaction mixture was stirred for 15 minutes at 0 ^{o}C and allowed to warm up to room temperature. The stirring was continued until nitroxide **17** disappeared. The progress of the reaction was monitored by TLC (silica gel, Et_2O/C_6H_{14} 1:5, visualization with 1% aq $KMnO_4$). Then the aqueous solution of ascorbic acid (0.3 g in 5 mL) was added in one portion and the resulted mixture was extracted with EtOAc (2 × 5 mL). The organic layer was dried with Na_2SO_4. After evaporation of the solvent, the crude residue was purified via column chromatography (silica gel, Et_2O/C_6H_{14} 1:5).

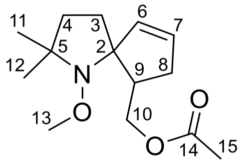

Yield: 40%, yellow oil.

IR (neat) ν, cm^{-1}: 3054, 2964, 2937, 2808, 1741, 1616, 1458, 1377, 1363, 1319, 1236, 1174, 1087, 1047, 1031, 970, 891, 858, 831, 748, 730, 723, 700, 661, 605, 549.

^{1}H NMR (600 MHz, $CDCl_3$): 1.07 and 1.18 (s both, 2×3H)(C(11)H_3 and C(12)H_3); 1.56 (dd, J_{d1}=6.5 J_{d2}=8.5, 2H)(C(4)H_2); 1.77 (dd, J_{d1}=6.5 J_{d2}=13.0, 1H)(C(3)H_2); 1.93 (dd, J_{d1}=8.5 J_{d2}=13.0, 1H)(C(3)H_2); 2.02 (s, 3H)(C(15)H_3); 2.11 (dddd, J_{d1}=1.9 J_{d2}=2.6 J_{d3}=8.1 J_{d4}=15.3, 1H)(C(8)H_2); 2.26 (dddd, J_{d1}=6.2 J_{d2}=7.7 J_{d3}=8.1 J_{d4}=8.3, 1H)(C(9)H); 2.36 (dddd, J_{d1}=1.7 J_{d2}=2.9 J_{d3}=7.7 J_{d4}=15.3, 1H)(C(8)H_2); 3.49 (s, 3H)(C(13)H_3); 4.14 (dd, J_{d1}=8.3 J_{d2}=10.9, 1H)(C(10)H_2); 4.45 (dd, J_{d1}=6.2 J_{d2}=10.9, 1H)(C(10)H_2); 5.64 (ddd, J_{d1}=1.7 J_{d2}=2.6 J_{d3}=6.1, 1H)(C(6)H=); 5.86 (ddd, J_{d1}=1.9 J_{d2}=2.9 J_{d3}=6.1, 1H)(C(7)H=)

^{13}C NMR (150 MHz, $CDCl_3$): 20.94 and 22.33 (C(11)H_3 and C(12)H_3), 29.79 (C(15)H_3), 34.35, 35.50, 35.60 (C(3)H_2, C(4)H_2, C(8)H_2), 47.41 (C(9)H), 62.78 (C(13)H_3), 64.78 (C(5)), 65.97 (C(10)H_2), 78.16 (C(2)), 131.12 (C(7)H=), 135.30 (C(6)H=), 171.06 (C(14)=O).

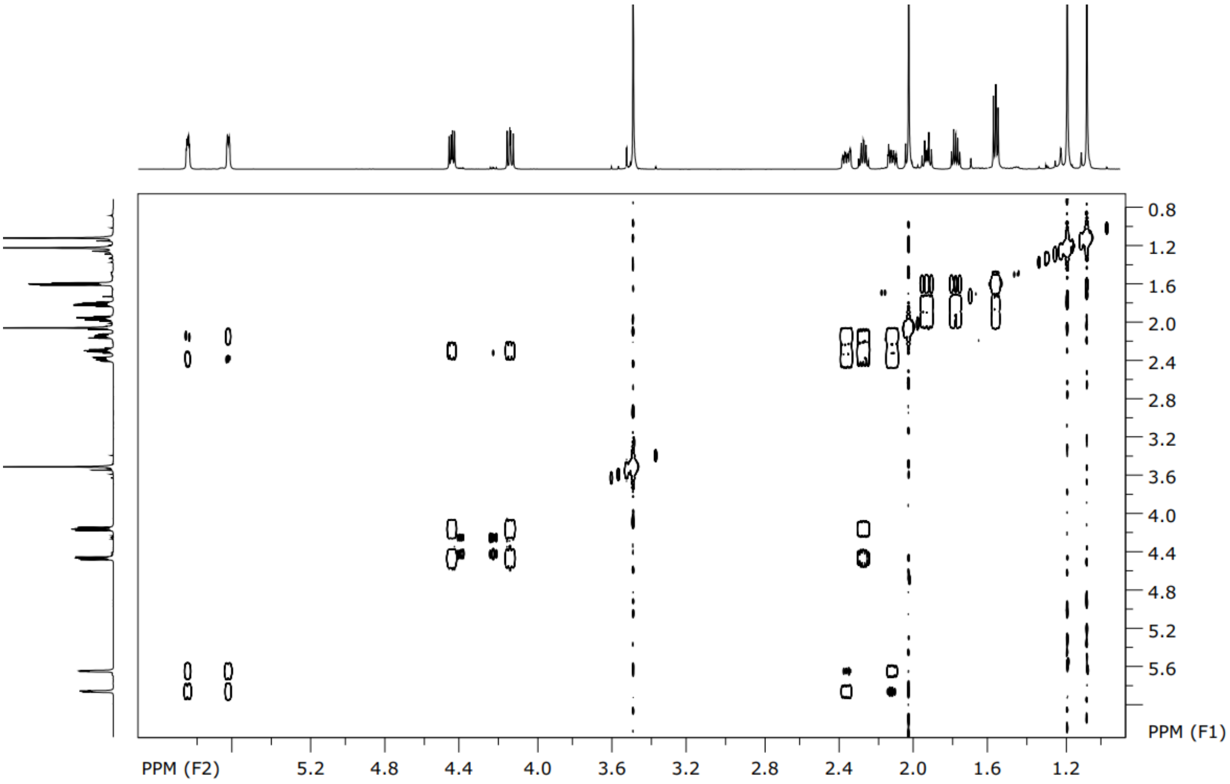

Figure S3: COSY ^{1}H-^{1}H of alkoxyamine **18**.

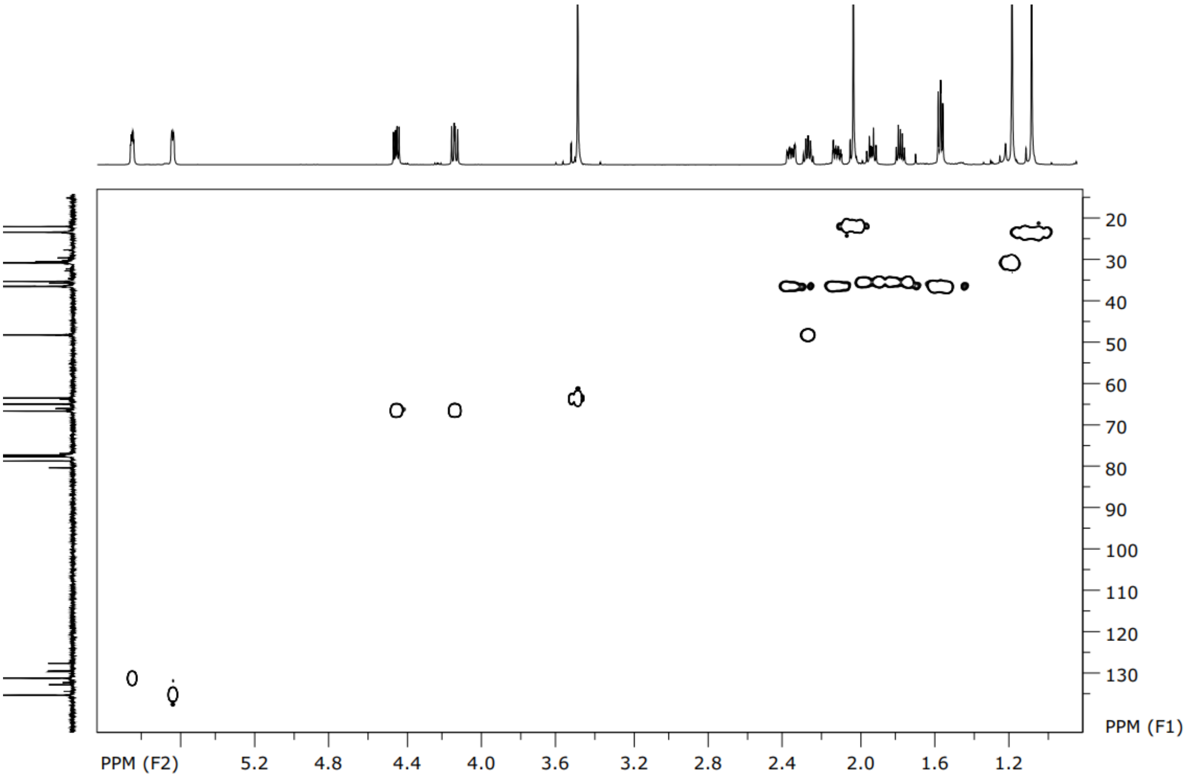

Figure S4: HSQC ^{1}H-^{13}C of alkoxyamine **18**.

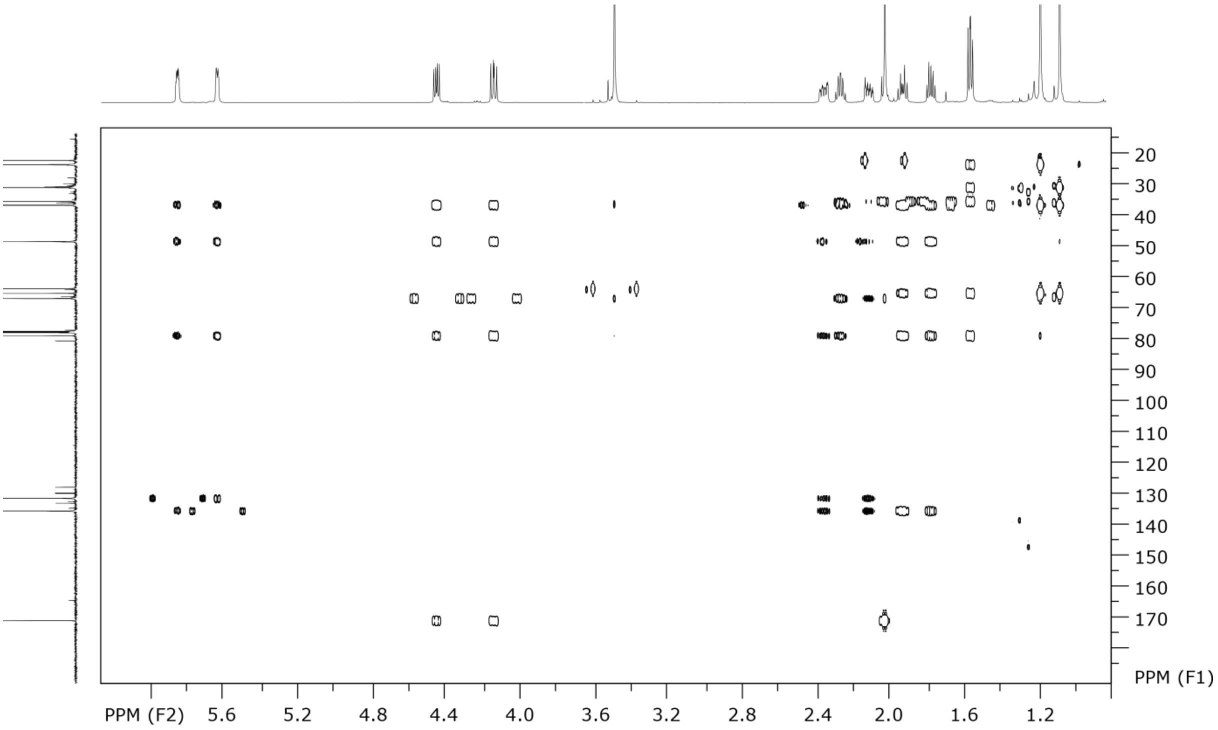

Figure S5: HMBC ^{1}H-^{13}C of alkoxyamine **18**.

EPR experiments were performed on CW EPR X-band spectrometer Bruker ER-200D (9.87 GHz). **Water solutions.** Spectra were acquired in oxygen-free conditions (via bubbling with argon) using following settings: microwave power 10 mW, modulations amplitude 0.1 or 0.2 mT; time constant 100 ms; conversion time 50.12 ms. Spectral parameters of radicals, a_N and ΔB_{p-p} were calculated using home-made software, *g*-factors were calculated using LiF:Li (g = 2.002293) as a reference. **Toluene solutions.** Solution of nitroxide (0.1-0.2 mM) in toluene was degassed by 3-4 freezing–pumping–thawing cycles. Spectrometer settings were as follows: frequency 9.87 GHz; microwave power 2.0 mW; modulation frequency 100 kHz; modulation amplitude 0.01 mT; conversion time 20 ms; number of points 2048; number of scans 8. EasySpin software was used for simulation of spectra.

For kinetic measurements stock solutions of ascorbic acid (1 M) and of GSH (100 mM) in PBS buffer (1 mM) were prepared and pH was adjusted to 7.4-7.5 with NaOH

Nitroxides were dissolved in phosphate-citrate-borate buffer, 3×50 mM, pH=7.5) and diluted to a concentration of 0.2-0.3 mM. All solutions were deoxygenated with argon, carefully and quickly mixed in a small tube and placed into EPR capillary (50 μL). Oxygen-free conditions were kept permanently. Capillary was sealed from both sides and placed into EPR resonator. The decay of amplitude of low field component of the EPR spectrum was followed in kinetics measurements. Kinetics of decay was fitted with monoexponential function to calculate the first order rate constants. Then these constants were divided into the concentration of ascorbic acid to calculate the second-order reaction constants.

Calculation of Lipophilicity. Lipophilicity was calculated as the partition coefficient of radical in the water-octanol mixture. For this purpose, the amplitude of the EPR spectrum of radical in water was recorded at different portions of added octanol. After addition of octanol, the mixture was extensively shaked, then shortly centrifuged to separate the water-octanol fractions.

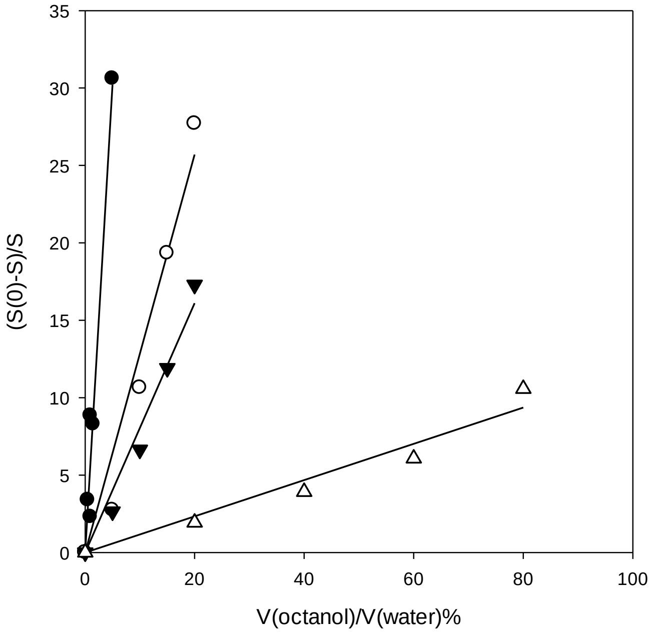

Figure S6: Calculation of lipophilicity of radicals. S – amplitude of radical in water at different portions of octanol, S0 – signal in water without octanol. The absolute slope

of the dependences are partition/lipophilicity coefficients that derives from theoretical equation: $(S_0-S)/S=k*V_{octanol}/V_{water}$.

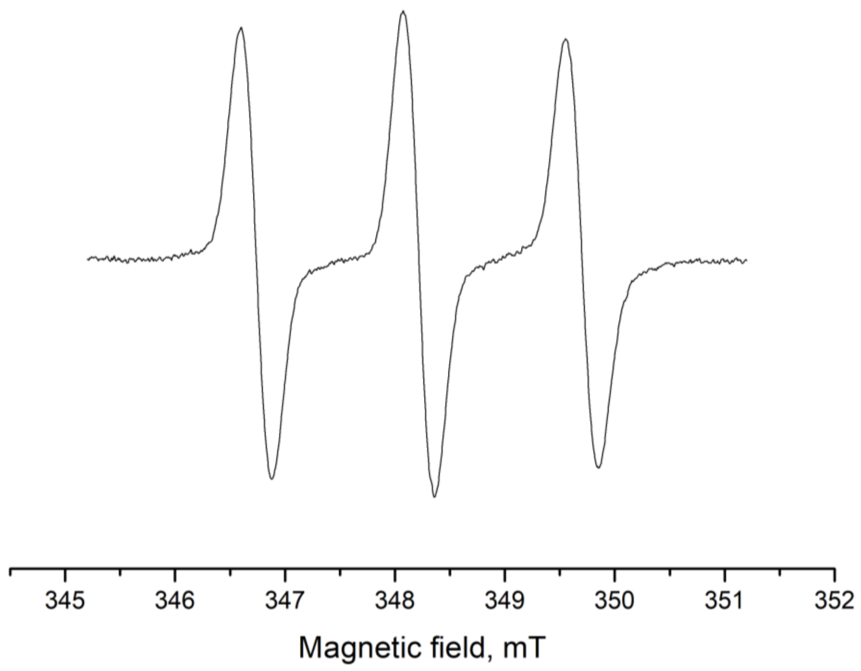

Figure S7: EPR spectra of radical **1** (water solution).

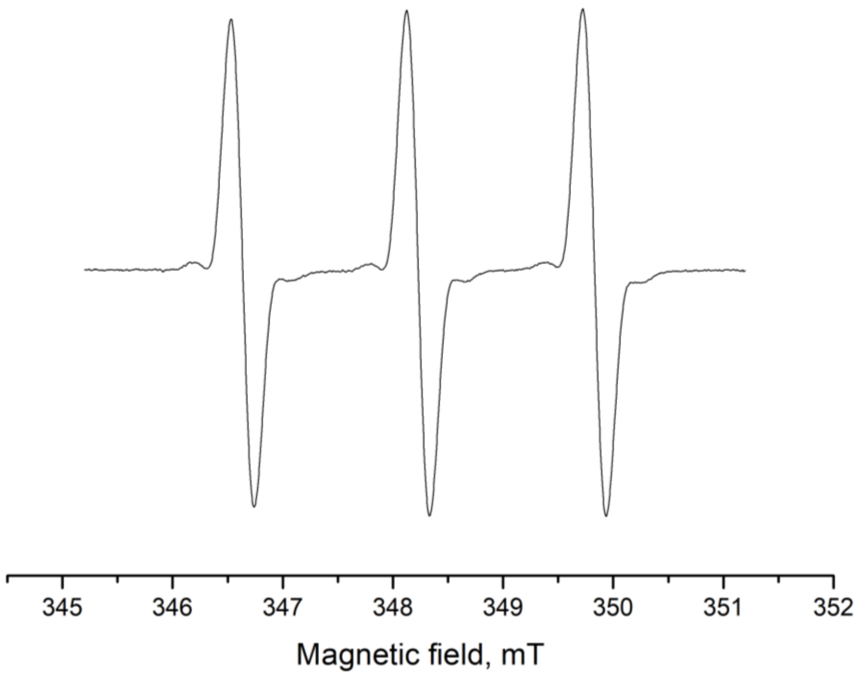

Figure S8: EPR spectra of radical **12a** (water solution).

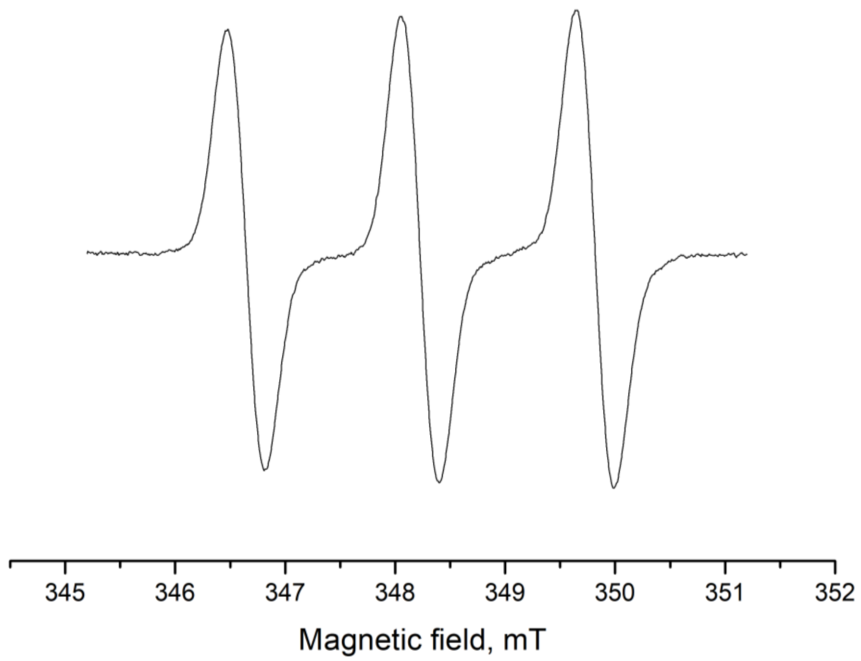

Figure S9: EPR spectra of radical **12b** (water solution).

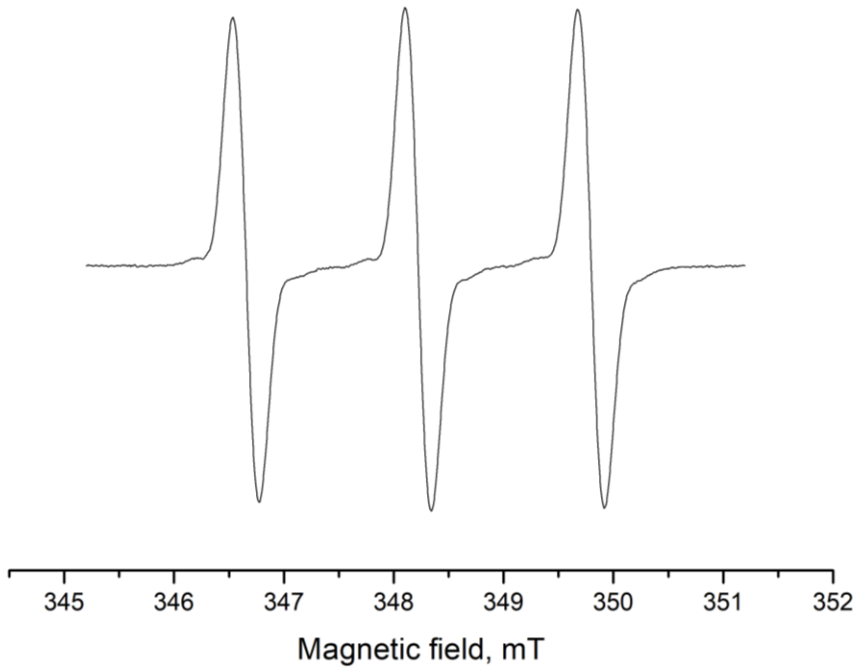

Figure S10: EPR spectra of radical **12c** (water solution).

Table S1: Hyperfine coupling constants, a_N (mT).

compound	Toluene solution	Water solution
**11a**	1.413	n/a
**11b**	1.405	1.580
**11c**	1.400	1.565
**12a**	1.450	1.595
**12b**	1.440	1.586
**12c**	1.440	1.570

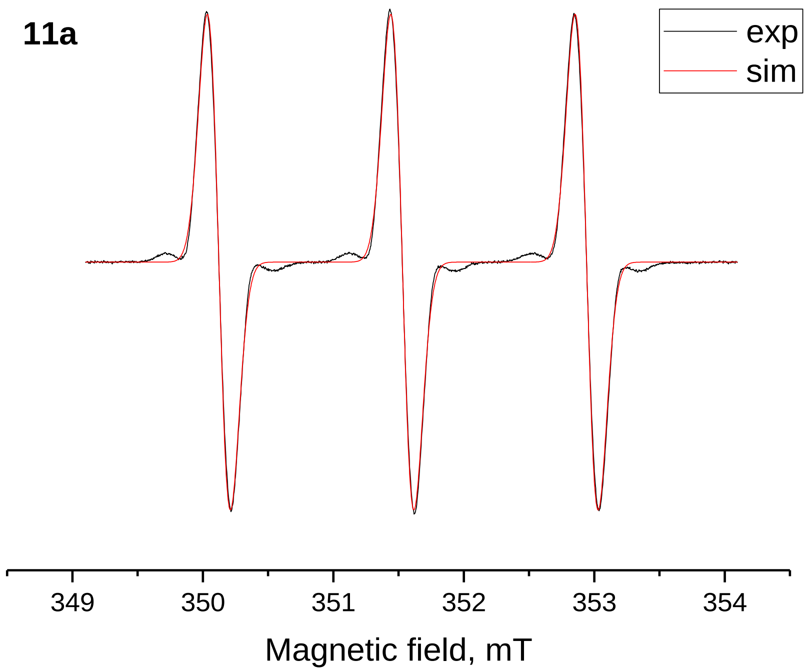

Figure S11: EPR spectra of radical **11a** (toluene solution).

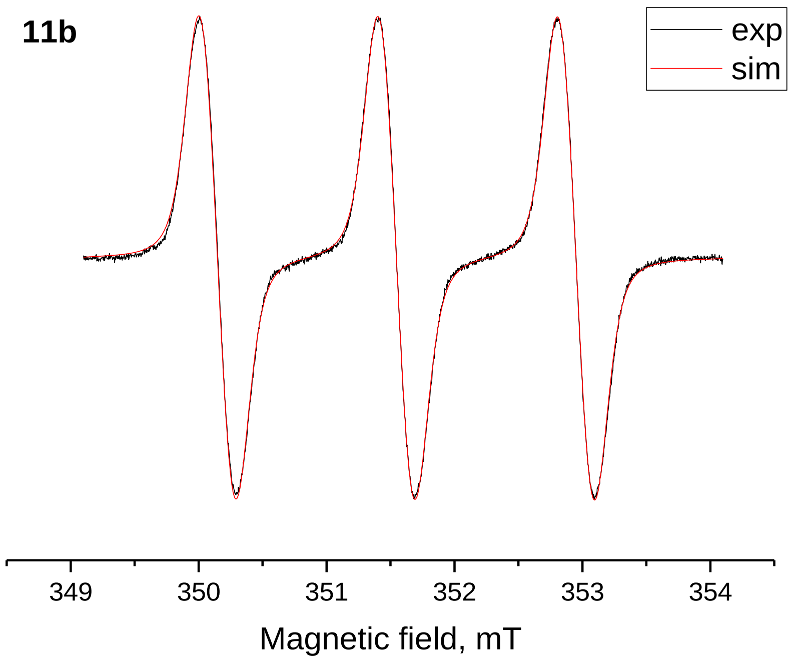

Figure S12: EPR spectra of radical **11b** (toluene solution).

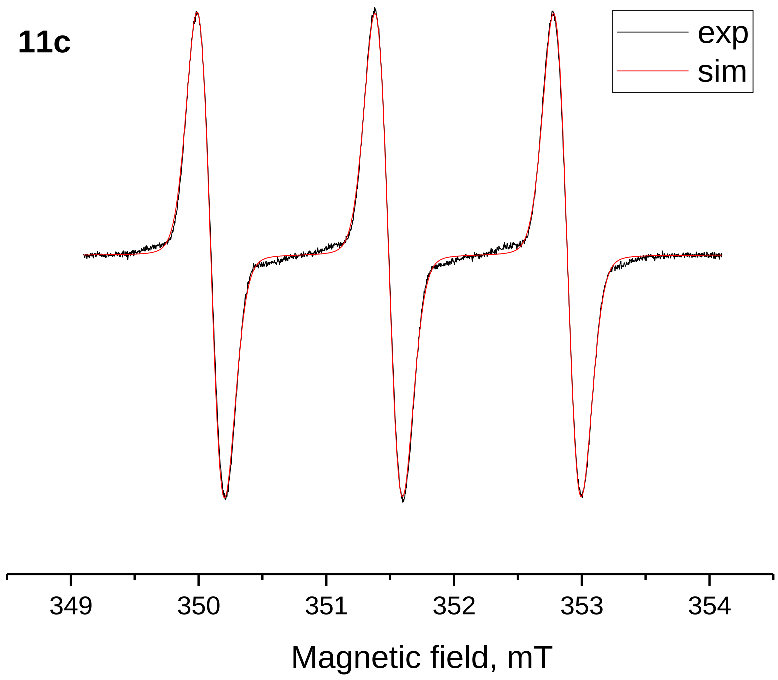

Figure S13: EPR spectra of radical **11c** (toluene solution).

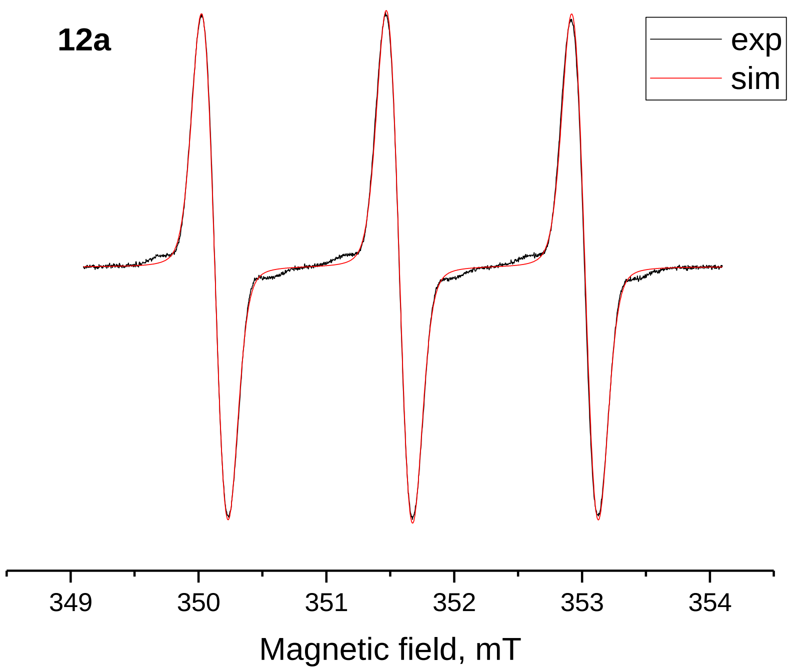

Figure S14: EPR spectra of radical **12a** (toluene solution).

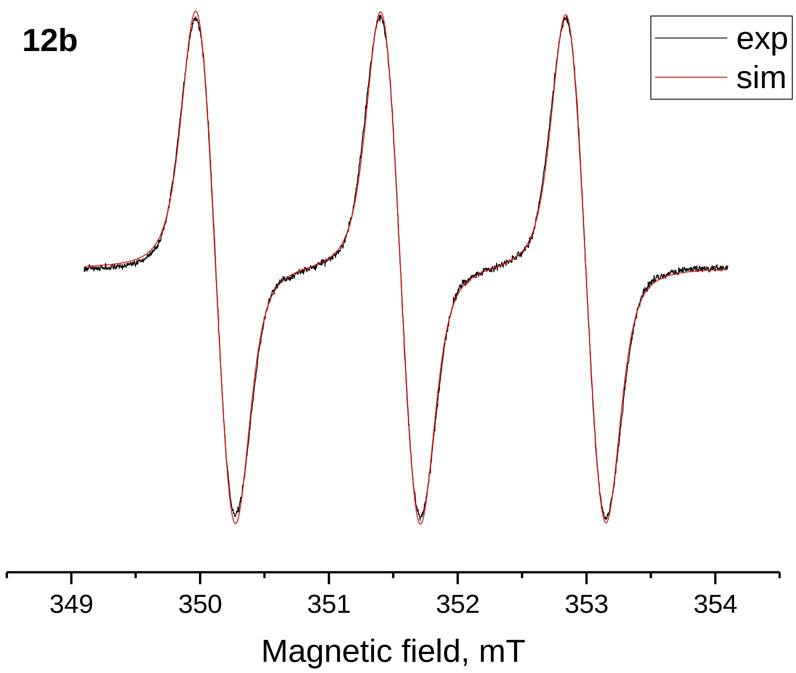

Figure S15: EPR spectra of radical **12b** (toluene solution).

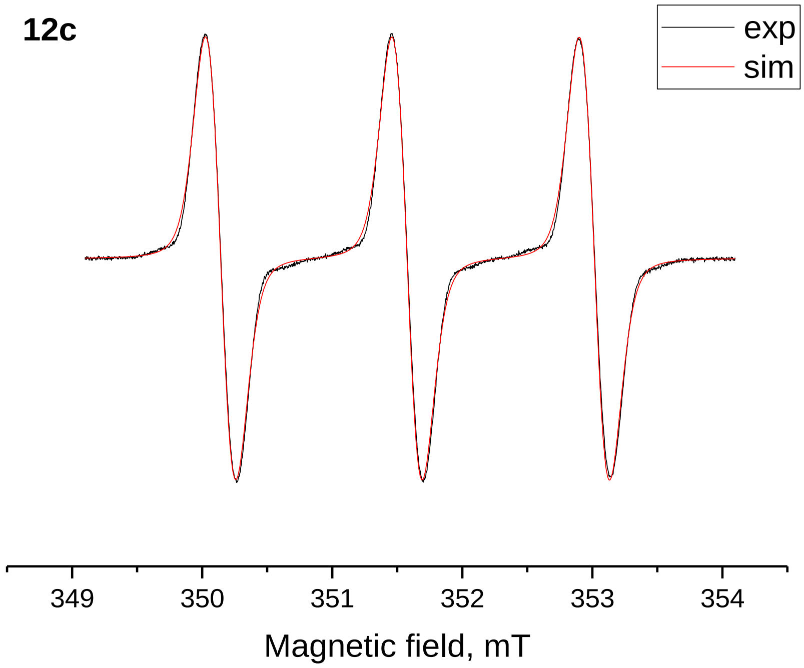

Figure S16: EPR spectra of radical **12c** (toluene solution).

References:

1. Haire, D. L.; Janzen, E. G. *Can. J. Chem*. **1982**, *60*, 1514-1522. doi: 10.1139/v82-220

2. Turner, M. J.; Rosen, G. M. *J. Med. Chem*. **1986**, *29*, 2439-2444. doi: 10.1021/jm00162a004

3. Corey, E. J.; Estreicher H. *Tetrahedron Lett.* **1980**, *21*, 1117–1120. doi: 10.1016/S0040-4039(01)83928-9

4. Chen, Yao-Jung; Wang Chi-Ying; Lin Wen-Yuan *Tetrahedron*, **1996**, *52 (41)*, 13181–13188. doi: 10.1016/0040-4020(96)00785-5

5. Matyjaszewski, K.; Woodworth, B. E.; Zhang, X.; Gaynor, S. G.; Metzner, Z. *Macromolecules*, **1998**, *31*, 5955–5957. doi: 10.1021/ma9807264

6. Schoening, K.-U.; Dichtl, A.; Seyfried, M. *Synlett*, **2008**, *12*, 1877–1881. doi:10.1055/s-2008-1078526
